# Bioactive scaffolds for tissue engineering: A review of decellularized extracellular matrix applications and innovations

**DOI:** 10.1002/EXP.20230078

**Published:** 2024-04-11

**Authors:** Juan Liu, Qingru Song, Wenzhen Yin, Chen Li, Ni An, Yinpeng Le, Qi Wang, Yutian Feng, Yuelei Hu, Yunfang Wang

**Affiliations:** ^1^ Hepato‐Pancreato‐Biliary Center Beijing Tsinghua Changgung Hospital School of Clinical Medicine Tsinghua University Beijing China; ^2^ Key Laboratory of Digital Intelligence Hepatology Ministry of Education School of Clinical Medicine Tsinghua University Beijing China; ^3^ Clinical Translational Science Center Beijing Tsinghua Changgung Hospital Tsinghua University Beijing China; ^4^ College of Chemistry and Life Sciences Beijing University of Technology Beijing China; ^5^ Institute of Smart Biomedical Materials School of Materials Science and Engineering Zhejiang Sci‐Tech University Hangzhou People's Republic of China; ^6^ Department of Hepatobiliary and Pancreatic Surgery The First Hospital of Jilin University Jilin University Changchun China

**Keywords:** 3D scaffolds, decellularization, extracellular matrix, native microenvironment, tissue engineering

## Abstract

Decellularized extracellular matrix (dECM) offers a three‐dimensional, non‐immunogenic scaffold, enriched with bioactive components, making it a suitable candidate for tissue regeneration. Although dECM‐based scaffolds have been successfully implemented in preclinical and clinical settings within tissue engineering and regenerative medicine, the mechanisms of tissue remodeling and functional restoration are not fully understood. This review critically assesses the state‐of‐the‐art in dECM scaffolds, including decellularization techniques for various tissues, quality control and cross‐linking. It highlights the functional properties of dECM components and their latest applications in multiorgan tissue engineering and biomedicine. Additionally, the review addresses current challenges and limitations of decellularized scaffolds and offers perspectives on future directions in the field.

## Graphical abstract

We conducted a critical evaluation of decellularization techniques, quality control, cross‐linking, and modification methods for dECM scaffolds derived from different sources. The functional properties of dECM components as well as their recent applications in multi‐organ tissue engineering and biomedicine were thoroughly reviewed. This work aims to discuss and summarize the current challenges and limitations of acellular scaffolds while also outlining future directions.

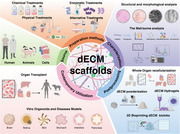

## 1 INTRODUCTION

Tissue engineering harnesses biomedical techniques to reconstruct organs and tissues, focusing primarily on the use of biocompatible materials such as synthetic polymers and natural/synthetic hydrogels, alongside physicochemical stimuli like bioactive agents and mechanical stresses to facilitate cellular augmentation.^[^ ^1^, ^2^ ^]^ A critical endeavor within this field involves the development of biomimetic scaffold systems that accurately replicate the intricate microenvironment of native tissues and organs.^[^ ^3^ ^]^ Traditional scaffold fabrication methods, including particulate leaching, freeze‐drying, and electrospinning, produce three‐dimensional porous structures that emulate the tissue‐like microenvironment. Advanced techniques, notably additive manufacturing, enable the creation of structures with gradient porosity along the scaffold length, which enhances tissue interface engineering. Despite these advancements, engineered constructs frequently lack the complex microenvironment and natural extracellular matrix (ECM) components necessary for optimal regeneration.

The complex microenvironment of native tissues presents numerous challenges, particularly in reproducing in vivo cellular interactions and their functionality. The ECM's functional characteristics and structural components, such as collagen and proteoglycans, play a critical role in maintaining tissue homeostasis and promoting angiogenesis.^[^ ^4^, ^5^ ^]^ These characteristics make replicating 3D ultra‐structures using traditional methods and materials challenging.

Decellularized tissues serving as potential biological scaffolds, have garnered significant attention. The decellularization process entails the removal of cellular components while preserving or minimizing the loss of tissue or organ‐specific ECM characteristics and, in some instances, retaining vascular and neural networks and structures. The maintenance of intrinsic biochemical and biophysical cues within the natural ECM yields structural and chemical signaling cues that support cell adhesion, migration, proliferation, and differentiation.^[^ ^6^, ^7^, ^8^, ^9^ ^]^ Decellularized extracellular matrix (dECM) contains an abundance of tissue‐specific growth factors and signaling molecules, which play a pivotal role in the development of the cellular microenvironment niche and offer broad prospects for decellularized tissue biomaterials as natural, bioactive grafts to regulate and enhance tissue‐specific cellular behavior and foster regeneration.^[^ ^10^, ^11^, ^12^ ^]^

**FIGURE 1:**
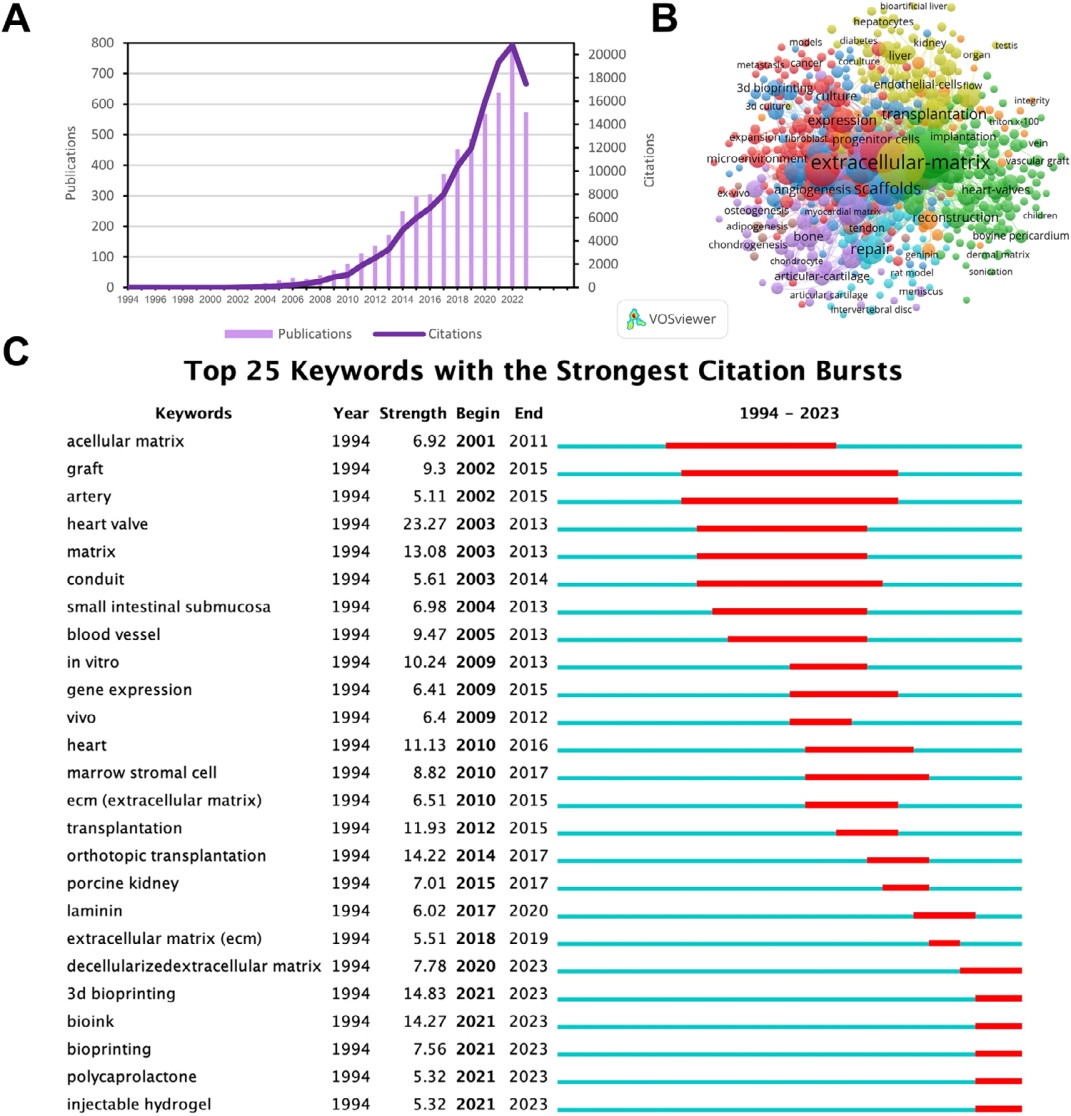
The development trend of dECM scaffolds. (A) Global trends and (B) research directions in publications and citations of decellularized extracellular matrix analysis in Web of Science from 1994 to 2023. (C) Visualization map of top 25 keywords with the strongest citation bursts from 1994 to 2023.

Over the past two decades, research interest in and advancements of decellularized biomaterials and grafts have significantly increased (Figure 1A). A Web of Science search for “Decellularized extracellular matrix” resulted in 5458 scholarly works, amassing a cumulative citation count of 132,705. These publications span a broad spectrum of tissue and organ types, such as the heart, skin, liver, and bone (Figure 1B). Furthermore, emerging technologies like 3D printing and injectable hydrogels have increasingly become prominent keywords in dECM research (Figure 1C). The application of decellularized scaffolds has rapidly diversified into areas including organ engineering, bioprinting, tissue repair and reconstruction, disease modeling, drug screening, and cell culture, presenting innovative solutions to donor shortages and limitations of in vitro models. This article offers an overview of the decellularization process, detailing methods for scaffold decellularization and crosslinking. Additionally, it reviews their applications in tissue engineering and biomedicine, addresses the challenges encountered in their preparation and application, and provides guidance for the judicious use of decellularized scaffolds in these domains to enable a wider spectrum of applications.

## 2 THE SOURCE OF DECM SCAFFOLDS

### 2.1 Human or animal organs/tissues source

Organ‐derived dECM scaffolds, obtained from specific organs or tissues, maintain the natural 3D structure by eliminating immunogenic cells and preserving the non‐immunogenic ECM. Following the process of decellularization, these organs/tissues yield dECM scaffolds that can then be seeded with specific cells to fabricate tissue‐engineered grafts. The availability of autologous and allogeneic tissues is limited. Xenotransplantation continues to be a viable solution for addressing human tissue shortages. Furthermore, the origin of tissues can influence the composition, degradation rate, and mechanical properties of dECM.^[^ ^13^ ^]^ Pig organs are more readily available and abundant compared to those of other animals.^[^ ^14^ ^]^ Given their robust reproductive capabilities and prolific offspring, pigs are traditionally the favored source of dECM for tissue and organ provision, though the risk of porcine endogenous retrovirus (PERV) transmission to humans persists.

To circumvent animal‐borne diseases and potential residual contamination and immunogenicity of xenogeneic dECM from animals, clinicians prefer human tissue for applications.^[^ ^15^, ^16^ ^]^ Human‐derived dECM can be sourced from cadavers, patients with diseased or damaged tissues and organs, and donations to human tissue biobanks. A diversity of human tissues and organs, including whole hearts, cartilage, ovarian tissue, adipose tissue, pancreas, kidneys, liver, skin, teeth, and lungs, have been successfully decellularized and analyzed the immunogenicity. For instance, Raghavan et al. investigated the development of an implant method to assess the surgical suitability of tendon grafts and discovered that the immunogenicity of decellular human tendons was suppressed following implantation into the subcutaneous tissue on the backs of immunologically normal rats.^[^ ^17^ ^]^ Xenografts, including porcine and bovine pericardium replacing autologous pericardium, have undergone extensive investigation, and while fixation with glutaraldehyde during transplantation diminishes graft immunogenicity, it may also result in accelerated calcification and cytotoxic effects attributable to fixation agents.^[^ ^18^, ^19^, ^20^ ^]^ Sackett et al. emphasize the significance of assessing the induction of immune responses following tissue replacement transplantation during allotransplantation using decellularized pancreatic tissue. They assessed the immunogenicity of hP‐HG by administering a neutralized pre‐gel solution into the backs of humanized mice. In vitro analysis revealed successful elimination of cellular and antigenic tissue components through decellularization treatment, and demonstrated low immunogenicity of human pancreas‐derived hydrogels.^[^ ^21^ ^]^ Subsequently, Choi et al. posited that human placenta could serve a pivotal role as a dermal substitute for full‐layer wound healing, attributed to its anti‐inflammatory, antibacterial, and low immunogenicity biological properties.^[^ ^22^ ^]^ While excellent results have been reported in the literature concerning decellularization and in vitro recellularization of various human tissues/organs, further efforts are required in the methods of surgical implantation and the development of human grafts for highly complex organs (liver, kidney, or lung), primarily due to the challenge of reproducing their functional tissue structure in vitro.

### 2.2 Cell source

Cell‐derived dECM scaffolds are promising substitutes for tissue‐derived options, providing critical environments for cell functions. Cells in vitro secrete ECM, which post‐decellularization, becomes a medium supporting robust cell growth.^[^ ^23^, ^24^ ^]^ Such scaffolds are generated from primary cells, sourced directly from tissues without passaging, and cell lines. Primary cells are preferred in tissue engineering for their in vivo‐like phenotypes and for creating substrates that mimic natural microenvironments. Mesenchymal stem cells (MSCs) are particularly useful in this realm for their ECM deposition, resembling various tissues and modifiable by culture conditions, thus commonly used for cell‐derived ECM production.^[^ ^25^ ^]^ Nonetheless, cell‐derived dECM's mechanical properties can be inadequate. To overcome this, it can be reinforced with biomaterials like hydroxyapatite or biphasic calcium phosphate, improving its structural integrity.

## 3 PREPARATION METHODS OF DECM SCAFFOLDS 

**FIGURE 2:**
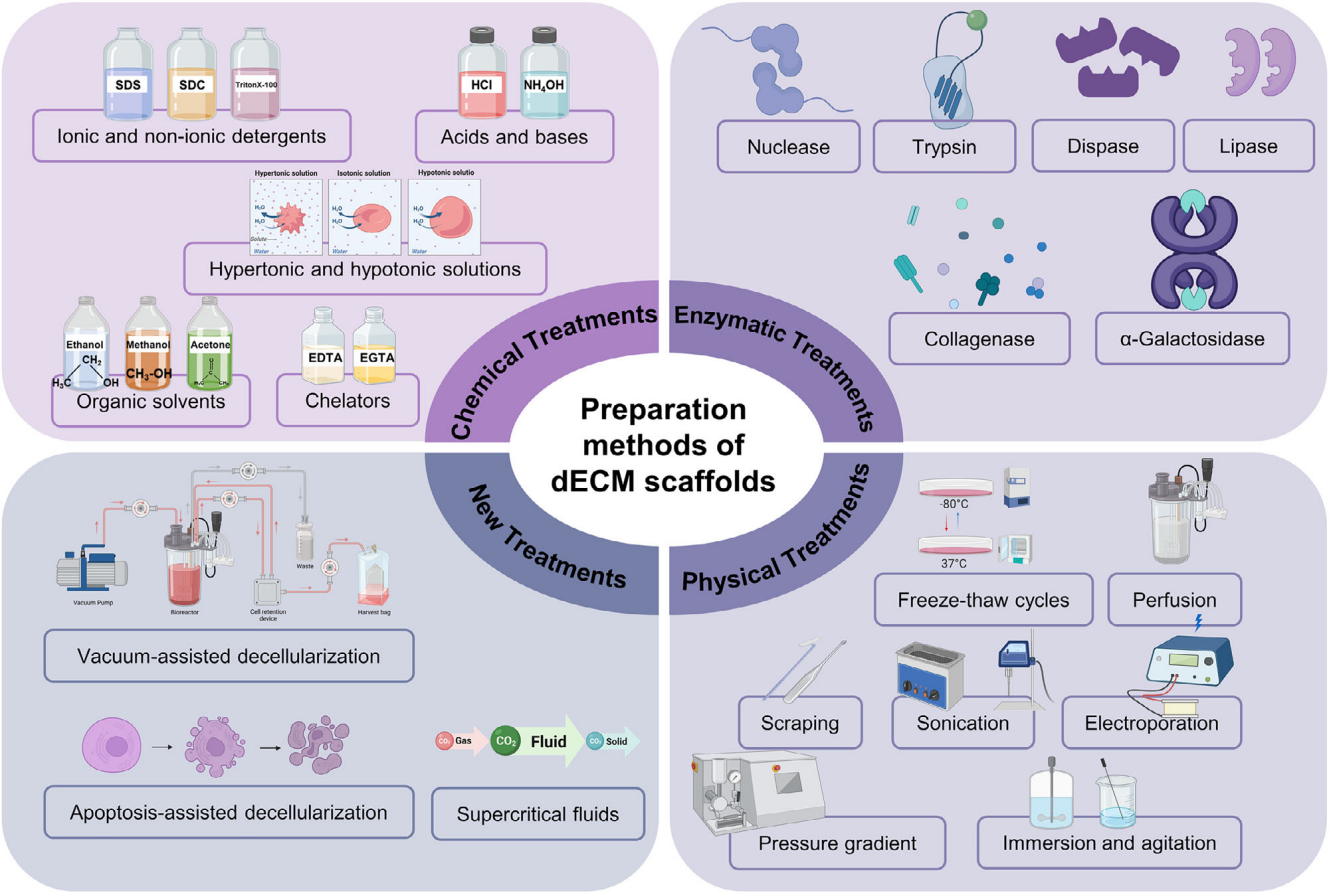
Preparation methods of dECM scaffolds. The preparation methods of dECM scaffolds mainly include chemical treatments, enzymatic treatments, physical treatments and new treatments. Chemical treatments: ionic and non‐ionic detergents (SDS, SDC, Triton X‐100, etc.), acids and bases solution (HCl, NH_4_OH, etc.), hypertonic and hypotonic solutions, organic solvents (ethanol, methyl alcohol, acetone, etc.), chelators (EDTA, EGTA, etc.). Enzymatic treatments: nuclease, trypsin, dispase, lipase, collagenase, α‐Galactosidase, etc. Physical treatments: freeze–thaw cycles, perfusion, mechanically scraping, sonication, electroporation, pressure gradient, immersion and agitation, etc. New treatments: vacuum‐assisted decellularization, apoptosis‐assisted decellularization and supercritical fluids (scCO_2_). Created with BioRender.com.

The pursuit of optimal decellularization resides in the complete removal of cellular constituents while meticulously conserving the inherent structure, composition, and biochemical and mechanical properties of the ECM. The absence of a universally accepted methodological standard for decellularization is largely attributed to the heterogeneity in factors such as species, age, anatomical positioning, and size of the source tissue. Numerous decellularization protocols have been developed, encompassing an array of physical, chemical, and enzymatic treatments, often in combination.^[^ ^26^, ^27^, ^28^ ^]^ Assessments of these strategies necessitate a hierarchical focus, with priority given to cellular and genetic material removal, followed by structural protein preservation.^[^ ^9^ ^]^ A comparative analysis of the mechanical attributes and ultrastructural characteristics of tissues or organs before and after treatment is indispensable. Emphasis is placed on the method's capacity to retain characteristics that are indispensable for successful tissue or organ reconstruction. Table 1 and Figure 2 summarize the chemical and enzymatic reagents, in conjunction with mechanical techniques, employed in decellularization processes.

**TABLE 1:** List of commonly used decellularization methods.

Sorts	Methods	Mechanisms	Advantage	Disadvantage	Applicability	Reference
Chemical treatments	Ionic detergents (SDS, SDC)	Perturb the phospholipid bilayers of cell membranes, resulting in cellular lysis	Disrupt a multitude of intracellular and intercellular interactions	More disruptive to the ECM due to substantial damage to collagen and glycosaminoglycan content	Entire organs and denser tissues	[9, 29]
	Nonionic detergents (Triton X‐100/200)	Solubilize cellular and nuclear membranes and denature proteins	Relatively mild effects on tissue structure. Remove cellular contents without disrupting collagen	Unable to denature proteins and remove cell nuclei, DNA, require combination with other methods	Entire organs	[9, 30]
	Zwitterionic detergents (CHAPS, SB‐10/16)	Exhibit properties of both ionic and nonionic detergents	Better protection of ECMs, a greater tendency to denature proteins	High residual DNA levels, additional washing steps to enhance efficiency	Blood vessel and nerve decellularization	[31, 32, 33]
	Acids (CH₃CO₃H, HCl)	Donate H^+^ or forming covalent bonds with electron pairs to disrupt the components and structure of ECM	Biocompatible, efficient in removing cells	A negative impact on ECM constituents and structures	Thin tissues	[34, 35, 36]
	Bases (NaOH, NH_4_OH, Na₂S)	Release OH^‐^ and react with acids to form salts, decellularizing tissues by denaturing chromosomal DNA and inducing cell dissolution	Efficient in removing cells and may have some bactericidal effect	Remove growth factors from the ECM and decrease mechanical properties by disrupting collagens	Thin tissues	[34, 37, 38]
	Hypertonic solutions	Due to the osmotic effects, cell lysis, cell dehydration and cell death	In protein removal, maintain the integrity of the basement membrane and the functional properties of tissues	Incapable of fully purging cellular remnants	Tendon and nerve tissues	[39, 40]
	Hypotonic solutions	Due to the osmotic effects, cell lysis, cell dehydration and cell death	In the extraction of nuclear material and DNA	The dispersion of antigens and tissue edema	Orthopedic and tendon tissues	[39, 41, 42]
	Organic solvents (Ethanol, Methanol, Acetone)	Aid in tissue decellularization by dehydration and lysis of cells	Efficient removal of lipids from tissue	Disrupt native ECM ultrastructure/microstructure, and may dehydrate tissues, causing tissue opacity	Blood vessels and other tissues	[9, 29]
Enzymatic treatments	Chelators (EDTA, EGTA)	Cause subtle disruption of protein−protein interactions by binding to divalent cations such as Ca^2+^ and Mg^2+^	Facilitate the removal of cellular components from tissues	Not effective cell removal and leave the cellular residues; coupled with either enzymes or detergents	Elastic cartilage and lung tissues	[9, 29, 43]
	Trypsin	The cleavage of the peptide bonds on the carbon side of carboxyl‐side of arginine and lysine, resulting in the separation of cellular components from ECM	More effective to disrupt the ultrastructure, complete cell and cytoplasm removal, preservation of gag content	Alterations in mechanical properties	Vascular tissues	[43, 44, 45, 46]
	Nuclease (Benzonase/DNase)	Catalyze the hydrolysis of internal and terminal bonds in ribonucleotide or deoxyribonucleotide chains, resulting in the fragmentation of RNA or DNA sequences	Enable DNA removal while preserving proteins	Alter ECM structure, compromise mechanical stability, and the loss of ECM components	Nerve and corneal tissues	[47, 48, 49]
	Other enzymes (Dispase/Lipase/Collagenase/α‐Galactosidase)	Provide high specificity for removal of cellular residues and efficient cell disruption	Exhibit high specificity in removing cells or undesirable ECM remnants	Disrupt the native ECM structure, compromise intrinsic mechanical strength	Amniotic membrane and corneal tissues	[50, 51, 52, 53]
Physical treatments	Electroporation	Apply microsecond electrical pulses to tissues, form nanopores to disrupt cellular homeostasis and cell death	Minimize thermal damage and enable recellularization, preserve ECM structure and integrity	Limited by probe size	Tissues of a specific size	[29, 54]
	Freeze–thaw cycles	Decellularization process formed intracellular dice crystals promoting cell lysis and detachment	Lyse the cells efficiently and reduce chemical lysants. Protect the ECM structure and integrity	Destroy the ECM ultrastructure, the removal efficiency is not high, the long processing time	Tendons, ligaments and nerves	[55, 56]
	Agitation	Destroy cells using energy generated by mechanical shock	Simple operation process	Not conducive to maintaining the structure and continuity of ECM		[9]
	Immersion	Put the tissues into the decellularization agents	Low requirement for organizational integrity	Longer time spending, inadequate removal of DNA, loss of ECM constituents and ultrastructure, or function of the scaffold in vivo	More tissues	[57]
	Perfusion	Deliver decellularizing agents to cells and transporting cellular material from the tissue	Shorter time spending, preserve whole organ ECM structures. Remove DNA completely. The well retained ECM constituents and ultrastructure	High requirement for organ integrity	Large organs (porcine heart, lung), and fewer organs	[57]
	Pressure gradient	Disrupt the cellular membrane by pressure gradients	Reduce operating time	Requiring extensive washing and rinsing steps to remove the residual components., damage the tiny structure and integrity of the ECM	Hollow tissues, such as blood vessels and the intestinal tract	[55, 58]
	Pressurization	Apply direct pressure to lyse cell	Reduce exposure time to harsh detergents	Expensive to operate, damage collagen and elastin fibers, alter ECM structure and mechanical properties		[26, 59]
	Scraping	Mechanical scraping cells	Effective for the removal of some visible tissue, do not have additional adverse effects on the uncontacted tissue parts	Incomplete or excessive removal, unable to ensure that the integrity of the ECM	The small intestine, bladder, and other tissues	[60, 61, 62]
	Sonication	Intense shock waves and shear stresses on the tissue membrane, resulting in membrane integrity alteration, membrane invagination, pore formation, and cell lysis	High cell removal efficiency	Not conducive to maintaining the integrity of the structure of the ECM	Small‐caliber vascular tissue, meniscus	[63, 64]
Alternative treatments	Vacuum‐assisted decellularization	Employs negative pressure to accelerate the decellularization	Combined with physical, chemical, or enzymatic treatments, significantly boost efficacy	Enlarge pore size and disrupt collagen bundles, compromise the mechanical strength of certain tissues	Tracheal tissues and large‐size enthesis	[65, 66, 67]
	Apoptosis‐assisted decellularization	Employs apoptosis inducers such as camptothecin and vinblastine to eliminate cellular components	Maximal preservation of nerve tissue structure	Not yet widely used	Complex structures like neural tissue or vascular systems	[39, 68, 69]
	Supercritical fluids (scCO_2_)	The low viscosity and high transport characteristics of detergents exposure lead to cell removal	Replace harsh chemicals to sensitive materials, preserve biochemical and mechanical properties	Damage to the native ECM ultrastructure	Corneal tissue and pulmonary arteries	[70, 71, 72]

### 3.1 Progress in decellularized scaffold preparation using chemical methods

Chemical decellularization utilizes surfactants and reagents to dissolve intercellular bonds, thereby facilitating cell removal.^[^ ^9^, ^29^ ^]^ This technique employs surfactants, acids, and alkalis.^[^ ^55^ ^]^ Surfactants lyse cells by disrupting the membrane phospholipid bilayers and are categorized into ionic, non‐ionic, or amphoteric types based on their charge. Agents such as peracetic acid and sodium hydroxide break down cell membranes and nucleic acids via charge interactions. Furthermore, hyperosmotic and hyposmotic solutions are utilized to lyse cells and promote cell detachment via osmotic pressure.

#### 3.1.1 Surfactants

During the fabrication of decellularized scaffolds, ionic surfactants such as SDS and SDC play a pivotal role. These surfactants disrupt various cellular interactions, effectively eliminating cells and genetic material from tissues.^[^ ^64^ ^]^ However, these surfactants may compromise the ECM's integrity and pose challenges in removal, potentially leading to toxicity in subsequent recellularization efforts.^[^ ^33^ ^]^ SDS, while highly effective for whole organ decellularization and preserving tissue ultrastructure and vascular integrity, can damage key proteins and signaling molecules, thus altering mechanical properties and necessitating thorough rinsing.^[^ ^9^, ^40^, ^72^ ^]^ To mitigate these effects, non‐ionic surfactants such as Triton X‐100 are utilized after SDS application to minimize damage and preserve tissue mechanics.^[^ ^26^, ^73^ ^]^ However, non‐ionic surfactants alone frequently fail to completely remove nuclear material, necessitating additional steps. SDC enhances scaffold biocompatibility, yet may lead to DNA aggregation. The combination of SDC with DNase I degrades DNA across various tissues, though extensive rinsing is required to mitigate immunogenicity.^[^ ^31^ ^]^ CHAPS detergent preserves matrix proteins and tissue mechanics, however, it may be less effective at DNA removal compared to SDS, potentially necessitating additional washes.^[^ ^74^ ^]^ The selection of surfactants for decellularized scaffolds entails balancing efficiency, ECM preservation, and compatibility with recellularization.

#### 3.1.2 Acid and base solutions

In the preparation of decellularized scaffolds, employing acids and bases is essential for the decellularization process, aiding in the breakdown of biomolecules and cellular components. Acids such as PAA, HCl, and acetic acid, via proton donation and covalent interactions, are capable of disrupting ECM components and structure.^[^ ^75^, ^76^ ^]^ However, they may also stiffen tissues and reduce collagen, thereby affecting the ECM microstructure and necessitating a careful selection of type and concentration. Alkaline agents, including ammonium hydroxide and sodium hydroxide, denature DNA and induce cell lysis, yet may lead to tissue swelling, affecting GAG content and ECM mechanics. Prolonged exposure to alkaline conditions can also degrade growth factors, collagen, and GAGs, potentially triggering immune responses and fibrosis.^[^ ^77^ ^]^ Therefore, the careful selection of alkaline conditions is paramount in maintaining tissue integrity. Acids and bases play an integral role in scaffold preparation, yet their use must be judicious to preserve ECM function and structure, thereby enabling the production of effective, biocompatible scaffolds for regenerative medicine.

#### 3.1.3 Hypertonic and hypotonic solutions

Hypertonic and hypotonic solutions are employed in the fabrication of decellularized scaffolds for their osmotic effects, which induce cell lysis, dehydration, and apoptosis, thereby removing cellular content.^[^ ^29^ ^]^ Hypertonic solutions are particularly effective in protein removal, whereas hypotonic solutions excel at extracting nuclear material and DNA. These solutions can be readily rinsed out, thereby minimizing risks of cytotoxic residuals. A decellularization protocol incorporating 1 M NaCl with freeze/thaw cycles yielded non‐toxic tendon scaffolds compatible with hFPTs, presenting an alternative to more aggressive agents for human tendon reconstruction.^[^ ^53^ ^]^ Saline‐based solutions also effectively eliminate ECM residues, with hypertonic solutions preserving basement membrane and tissue functionality. For instance, NaCl treatment was more effective in preserving corneal clarity than treatments with SDS or nucleases. However, hypotonic solutions may lead to the dispersion of antigens and cause edema. Achieving complete cellular clearance often necessitates the inclusion of additional surfactants or enzymes, such as DNase, which can eliminate over 95% of DNA when used in conjunction with hypertonic washes.^[^ ^39^ ^]^ Thus, hypertonic and hypotonic solutions fulfill a crucial role in scaffold engineering, yet their effectiveness is amplified when combined with other agents.

#### 3.1.4 Organic solvents and chelators

Additionally, organic solvents such as ethanol, methanol, and acetone aid in tissue decellularization by dehydrating and lysing cells. However, due to their chemical properties and toxicity, some organic solvents such as hexane, commonly used in traditional decellularization methods, are also considered unsafe.^[^ ^78^ ^]^ Additionally, chelating agents such as ethylenediamine tetraacetic acid (EDTA) remove bivalent cations such as Ca^2+^, which play an important role in cell‐ECM adhesion, from the cell membrane binding site, thus separating the cell components from the ECM.^[^ ^79^, ^80^ ^]^ Additionally, chelating agents facilitate the removal of nuclear immunogens by consuming free bivalent cations that precipitate DNA on the ECM. However, the use of EDTA alone is not sufficient to obtain a suitable decellularized ECM scaffold.

### 3.2 Progress in decellularized scaffold preparation using enzymatic treatments

Enzymatic decellularization employs nucleases, proteases, and additional enzymes to break down cellular bonds and facilitate cell removal. Although these enzymes precisely remove cells and debris, achieving complete decellularization is challenging, and enzyme residues might impede recellularization and provoke immune responses. Trypsin, a serine protease, specifically cleaves peptide bonds, being particularly active at 37°C and pH 8. Initially used in decellularization for its efficacy in disrupting tissue ultrastructure and enhancing the penetration of other agents, trypsin may nevertheless impact ECM mechanics.^[^ ^43^, ^44^, ^45^, ^46^ ^]^ Optimal concentrations and application times of trypsin are crucial. Nucleases, including DNase, hydrolyze nucleotide bonds, thereby fragmenting DNA and RNA following cell lysis.^[^ ^29^, ^48^ ^]^ Frequently applied after detergents, they enhance tissue penetration and eliminate remaining nucleic acids. Combining DNase with detergents can achieve significant DNA clearance rates.^[^ ^38^, ^39^ ^]^ However, prolonged exposure to nucleases can modify ECM structure and compromise its mechanical stability.^[^ ^47^, ^49^ ^]^ Other enzymes, such as lipases, hydrolyze lipids, whereas dispersases and thermolysin target basement membrane proteins, with thermolysin proving less damaging to the ECM.^[^ ^50^ ^]^ Collagenase is utilized when preserving ultrastructure is not critical, and α‐galactosidase reduces immunogenic antigens without significantly impacting dECM remodeling. Enzymatic treatments, though specific, may disrupt ECM integrity and mechanical properties. On their own, they might not guarantee complete decellularization in thick tissues, and prolonged use poses risks of damaging recellularization and provoking immune responses. The combination of enzymes with chemical treatments and optimization of exposure times can enhance decellularization efforts while preserving dECM quality.

### 3.3 Progress in decellularized scaffold preparation using physical treatments

Physical decellularization methods, such as freeze‐thaw cycling, perfusion, and agitation, disrupt cell membranes and facilitate cell removal from tissues. Freeze–thaw cycling, resulting in intracellular ice formation, is widely used for ligaments and neural tissues, yet may leave behind membrane fragments. It is frequently combined with other methods to enhance ECM porosity, necessitating controlled temperature changes to minimize ECM damage. Perfusion decellularization, ideal for large tissues or organs, involves circulating decellularization agents through the natural vasculature, preserving the vascular network but requiring complex equipment.^[^ ^57^ ^]^ Immersion with agitation is suitable for smaller tissues lacking vascular networks, enhancing decellularization agent penetration via mechanical motion. Optimal agitation conditions are contingent upon tissue characteristics, and although simpler than perfusion, may result in more tissue damage due to limited diffusion.^[^ ^81^, ^82^ ^]^ Mechanical abrasion applies direct pressure to tissues, effectively removing cells from less dense ECM structures such as the lung. However, dense ECM tissues might experience integrity loss, necessitating careful application of pressure. Nonthermal irreversible electroporation (NTIRE) employs electrical pulses to create nanopores in cell membranes, leading to cell death but preserving ECM integrity. Its effectiveness is limited by probe size and might not achieve complete decellularization alone.^[^ ^29^, ^54^ ^]^ Physical methods frequently necessitate combination with chemical or enzymatic agents to ensure thorough decellularization and ECM preservation, particularly for tissues such as the bladder or small intestine.

### 3.4 Alternative decellularization agents and combinatory approaches

Recent advances in decellularization encompass vacuum‐assisted and apoptosis‐assisted techniques, offering increased efficiency and potential for complex tissues. Vacuum‐assisted decellularization, employing negative pressure to expedite the process, can be utilized in conjunction with other techniques to enhance outcomes.^[^ ^65^, ^66^, ^67^, ^83^ ^]^ However, there is a risk of altering the ECM's pore size and collagen structure, potentially affecting the mechanical integrity of tissues such as the pericardium. Apoptosis‐assisted decellularization utilizes agents such as camptothecin to induce cell death and is especially promising for neural tissue and vascular structures.^[^ ^39^, ^68^, ^69^ ^]^ Supercritical carbon dioxide (scCO_2_), when combined with ethanol treatment, effectively eliminates DNA and reduces phospholipid content in tissues, including the aorta, with minimal impact on mechanical properties and without necessitating lyophilization for ECM preparation and storage. To safeguard the ECM from protease damage during decellularization, serine protease inhibitors, including aprotinin, PMSF, and leupeptin, can be incorporated into the solutions.^[^ ^70^, ^71^, ^72^ ^]^ Buffering solutions to pH 7−8 and controlling the temperature and exposure duration can also aid in inhibiting protease activity. Despite their potential, these advanced techniques have yet to be widely implemented due to their complexity, remaining areas of active research. In an innovative approach, Chen et al. utilized electrospinning to fabricate decellularized spinal cord fibers (A‐DSCF) that preserve spinal cord ECM proteins and demonstrate enhanced mechanical properties, enzymatic resilience, and support for neural progenitor cell functions.^[^ ^84^ ^]^

To achieve successful decellularization, which involves removing cells while preserving the ECM, integrating multiple methods is often necessary. Hybrid approaches, combining physical, chemical, and biological techniques, are becoming increasingly common. For instance, chemical and physical methods can induce necrosis, releasing cellular contents into the ECM and necessitating thorough washing to remove debris.^[^ ^85^ ^]^ Duarte et al. demonstrated that the use of scCO_2_ with tri‐n‐butyl phosphate (TnBP) effectively reduced DNA content in decellularized porcine trabecular bone tissue, exemplifying the synergy between physical and chemical decellularization methods.^[^ ^86^ ^]^ Yüksel et al. achieved optimal decellularization of rat amniotic membranes through a protocol that included hypertonic media, UV exposure, deep freezing, and SDS washing, thereby creating a scaffold conducive to cell adhesion and survival.^[^ ^87^ ^]^ Shafiq's method for auricular cartilage decellularization integrated SDC, freeze–thaw cycles, DNase, and trypsin, which effectively removed cells and preserved collagen and biomechanical properties. This process was specifically tailored to minimize exposure to harsh chemicals and expedite decellularization.^[^ ^43^ ^]^ The selection of a decellularization strategy is dependent on tissue‐specific factors, including thickness, cellular density, and scaffold size. For instance, 1% SDS proved to be more effective than 1% Triton X‐100 in decellularizing human testicular tissue, attributed to SDS's superior penetration in thick tissues.^[^ ^88^ ^]^ However, SDS can lead to shrinkage and stiffening in dense matrices, such as tendons, highlighting its limitations for certain applications.^[^ ^40^ ^]^ Therefore, selecting the most suitable decellularization method necessitates careful consideration of the tissue characteristics and the desired outcome, balancing cell removal efficiency with ECM preservation.

### 3.5 Challenges and potential improvements in dECM preparation

The integration of various decellularization methods has indeed become a pivotal area of investigation as researchers strive to optimize the removal of cellular material while preserving the ECM. Challenges and potential improvements in decellularized scaffold preparation include: (a) Incomplete removal of cellular material—Residual nucleic acids and cellular components can provoke immune reactions or affect biological activity. Enhanced cell lysis, increased use of deoxyribonucleases, and biophysical methods such as ultrasound can improve cell residue elimination. (b) Preservation of ECM biological activity—The decellularization process can impair ECM components, thereby reducing their biological function. Optimizing conditions to minimize ECM damage, and potentially introducing bioactive factors, could aid in maintaining or restoring ECM functionality. (c) Structural integrity of scaffolds—Certain decellularization techniques can impair the scaffold structure, impacting biomechanical properties and cell adhesion. Selecting gentler cell removal methods and fine‐tuning enzymatic hydrolysis can prevent structural alterations. (d) Vascularization of scaffolds—Creating vascular networks within scaffolds is essential for tissue regeneration. Techniques such as pre‐fabricating microchannels or employing angiogenic growth factors can enhance vascularization. (e) Microbial contamination—Decellularization processes can introduce microbial contaminants. Sterile techniques and antibiotics can mitigate contamination risks. (f) Complexity and scalability—Certain decellularization methods are intricate and time‐consuming, impeding large‐scale production. Streamlining and optimizing these methods can lead to more streamlined production processes. Despite the variety of techniques documented, standardization and validation of protocols across different tissues and organs are necessary. Research gaps include determining optimal perfusion parameters, understanding the impact of detergent concentrations and pressure on ECM preservation, and addressing the challenge of cellular debris in vascular structures. An ideal decellularized scaffold should support cell adhesion, proliferation, and differentiation, meeting the structural requirements of complex organs.

## 4 CHARACTERIZATION FOR THE PREPARED DECM SCAFFOLDS

During tissue decellularization, it is critical to preserve the ECM's inherent ultrastructure and biochemical components. However, the fabrication of dECM from intact mammalian tissues involves multiple processing steps that can alter the composition, structural integrity, and features, which may compromise the biochemical and biomechanical properties of the resulting dECM‐based materials. Additionally, cell death during decellularization can release deleterious byproducts, which trigger inflammation and subsequent local/systemic reactions. Moreover, preserving essential ECM components—such as GAGs, laminins, and fibronectins (adhesion proteins), and growth factors, elastin, and collagen—is imperative, since they facilitate enhanced cellular infiltration into dECM‐based materials both in vitro and in vivo. An enhanced understanding of material properties requires performing both quantitative and qualitative analyses of ECM protein composition and distribution in dECM‐based materials. Table 2 presents the characterization techniques applied to dECM.

**TABLE 2:** List of dECM scaffolds characterization methods.

Sorts	Principle	Methods	Reference
Cell removal analysis	Remnant DNA/cell nuclei	HE (hematoxylin)	[89, 90, 91]
		Hoechst 33258	
		Fluorescent DAPI	
		PI	
		Picogreen	
		TUNEL assay	
		Spectrophotometric assays	
	Intracellular protein detection	Staining with fluorescent‐labeled phalloidin/rhodamine (actin)	[9, 92, 93]
		Immunocytochemistry (cytosolic proteins)	
		Immunohistochemistry (ECM proteins)	
		Masson's trichrome	
		Movat's pentachrome	
		Verhoeff's Van Gieson (EVG)	
		Safranin‐O	
		Oil Red O	
Compositional analysis	Non‐nucleic components	Eosin staining	[94]
	Collagens	Sirius red	[95, 96, 97]
		Azan staining	
		Hydroxyproline assay	
		Sircol kit	
	Proteoglycans/GAGs	Alcian blue staining	[90, 95–99]
		Toluidine blue staining	
		Dimethylmethylene blue (DMMB)	
		Blyscan kit	
	Glycoproteins‐Elastin	Ninhydrin Fastin	[97]
	Carbohydrates	Lectins staining	[100]
	ECM‐bound growth and secreted factors	ELISA‐based assays	[100]
	Other specific associated proteins	IHC or IF analysis with specific antibodies	[92]
	Matrisome protein profiles quantification	LC‐MS/MS、TOF‐SIMS、MALDI、mRNA microarrays	[101, 102]
Structural analysis	Morphological observation	Scanning electron microscopy (SEM)	[94, 103, 104]
	Ultrastructure detection	Optical microscopy	[94, 103]
		Transmission electron microscopy (TEM)	
		Atomic force microscopy (AFM)	
		Second harmonic imaging	
	Collagen, elastic fiber spatial alignment	Laser microscopy	[94, 105, 106, 107]
		X‐ray	
		Optical coherence tomography (OCT)	
		Raman imaging	

### 4.1 Structural and morphological analysis of dECM scaffolds 

**FIGURE 3:**
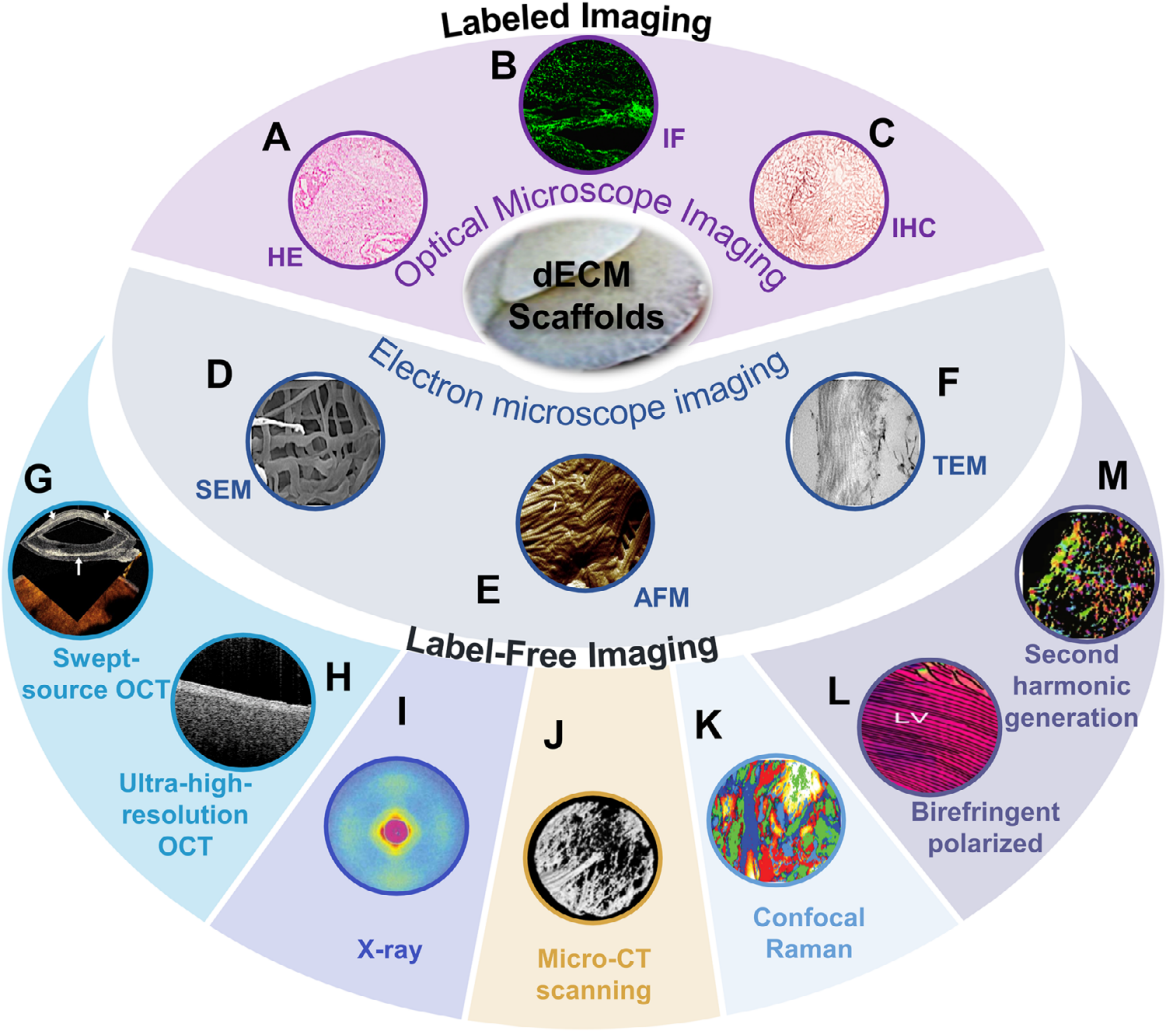
Structural and morphological analysis of the dECM scaffolds. (A) HE staining of decellularized rat liver scaffolds. Reproduced with permission.^[^ ^92^ ^]^ Copyright 2021, Elsevier. (B) Immunofluorescent staining of decellularized mouse liver scaffolds (collagen IV). Reproduced under Attribution‐Non Commercial‐No Derivs 4.0 International License (CC BY‐NC‐ND 4.0 DEED).^[^ ^113^ ^]^ Copyright 2023, The Authors. (C) Immunohistochemical staining of decellularized rat liver scaffolds. Reproduced with permission.^[^ ^92^ ^]^ Copyright 2021, Elsevier. (D) SEM imaging of decellularized rat liver scaffolds. Reproduced with permission.^[^ ^92^ ^]^ Copyright 2021, Elsevier. (E) AFM imaging of mouse lung ECM. Reproduced under CC BY‐NC‐ND 3.0 License.^[^ ^110^ ^]^ Copyright 2010, The Author(s), published by Elsevier. (F) TEM imaging of decellularized rat liver scaffolds. Reproduced with permission.^[^ ^92^ ^]^ Copyright 2021, Elsevier. (G) Swept‐source OCT (Optical Coherence Tomography) imaging of 3D bioprinted decellularized collagen grafts implanted into the cornea. Reproduced under the terms of the CC‐BY 4.0 license.^[^ ^114^ ^]^ Copyright 2019, The Authors. (H) Cross‐sectional UHR‐SD‐OCT image of membranous septum imaged from left ventricular aspect of a porcine heart. Reproduced with permission.^[^ ^115^ ^]^ Copyright 2022, Optica Publishing Group. (I) Wide‐angle synchrotron X‐ray scattering pattern from the central cornea. Reproduced with permission.^[^ ^111^ ^]^ Copyright 2004, Elsevier. (J) Representative 3D reconstructed micro‐CT images 8 weeks after the implantation of SIS‐based scaffolds. The new bone formation ratio. Reproduced under the terms of the Creative Commons Attribution License (CC BY).^[^ ^116^ ^]^ Copyright 2022, The Authors. (K) Confocal Raman microscopy analysis of ECM derived from decellularized bovine endometrium. Reproduced under the terms of the Creative Commons Attribution License.^[^ ^107^ ^]^ Copyright 2021, Simsa et al. (L) Birefringence polarization imaging of healthy rat hearts. Reproduced under the terms of the Creative Commons Attribution License (CC BY).^[^ ^108^ ^]^ Copyright 2021, The Author(s). (M) Second harmonic generation imaging of alcoholic cirrhosis in patients. Reproduced under the terms of the Creative Commons Attribution License (CC BY).^[^ ^109^ ^]^ Copyright 2023, The Authors.

The structure and morphology of decellularized scaffolds are critical for their applications in tissue engineering. Structural and morphological analyses are conducted using optical microscopy, histological staining, SEM, and TEM (Figure 3). Histological and immunohistochemical staining are commonly used image‐based techniques. However, the fixation, labeling, and processing steps may be destructive, potentially distorting cellular expression and tissue architecture, and tend to provide information for a limited tissue area.^[^ ^94^ ^]^ Standard optical microscopy, which is frequently employed for structural and morphological analysis, permits direct scaffold observation; however, its lower resolution restricts observation of microstructural details.^[^ ^94^ ^]^ Microscopic techniques are principally utilized to examine fiber alignment and pore architecture in decellularized scaffolds. The acquisition of paraffin or frozen sections and subsequent immunostaining determine the collagen type distribution in decellularized scaffolds. Immunostaining both native and decellularized tissue sections with type IV collagen antibodies, coupled with DAPI counterstaining, is used to visualize cell nuclei and confirm decellularization efficacy and ECM component preservation.^[^ ^93^ ^]^ An absence of cell nuclei and the presence of type IV collagen indicate a complete decellularization and preservation of ECM components. We report the absence of cell nuclei and the preservation of ECM components in liver‐derived dECM sections, as demonstrated by immunostaining with antibodies against Collagen I, III, V, IV, VI, Nidogen, Laminin, Fibronectin, and Perlecan, followed by DAPI counterstaining.^[^ ^92^ ^]^ Image‐based analysis serves as a platform to qualitatively confirm effective decellularization and the preservation of tissue‐specific ECM components, which may facilitate tissue regeneration and constructive remodeling.

Advanced techniques have been developed to alter the optical pathway, thereby enhancing the quality, resolution, and contrast of specimen images. Polarized microscopy facilitates the quantitative analysis of collagen organization.^[^ ^94^ ^]^ The orientation of collagen fibers in both healthy and diseased tissues is vividly highlighted by birefringent polarized images, indicating that this technique is beneficial for detecting subtle changes in collagen structure during decellularization.^[^ ^108^ ^]^ Second harmonic generation (SHG) imaging delivers images with exceptional spatial resolution and contrast, which are crucial for visualizing tissue architecture.^[^ ^94^, ^109^ ^]^ Jamaluddin et al. were the first to use confocal Raman imaging to detect components such as collagen, glycosaminoglycans (GAGs), and amino acids in decellularized scaffolds from bovine and human endometrium.^[^ ^107^ ^]^ This non‐invasive method obviates the necessity for tissue staining and further processing in qualitative imaging.

Electron microscopy represents an analytical technique that is primarily utilized to provide localized information on tissue architecture. Unlike optical microscopy, electron microscopy affords higher resolution, revealing intricate ultrastructural features.^[^ ^100^ ^]^ TEM elucidates ultrastructural details of tissues, including fibril diameter, spacing, and collagen fibril organization, establishing it as a critical technique for an in‐depth understanding of native tissue architecture.^[^ ^94^ ^]^ SEM, which probes sample surfaces, provides information on their topology and morphology. Although SEM possesses lower resolution than TEM, it is capable of determining the morphology of sample surfaces, evaluating 3D stratified structures in native and regenerative tissues, and imaging larger areas. For instance, SEM imaging demonstrates that collagen fiber alignment in decellularized bovine tendon is significantly distorted compared to the uniform and directionally stacked fibers in native tissues.^[^ ^94^ ^]^ Upon magnification, the preserved striation patterns and integrity of collagen fibers are substantiated, as further evidenced by immunostaining with type I collagen antibodies. However, the critical point drying technique for SEM sample preparation is linked to varying degrees of sample shrinkage and may necessitate harsh fixatives.^[^ ^94^ ^]^ In a study on rat renal decellularized scaffolds, TEM and SEM revealed that after 17 h of perfusion with SDS, the whole kidney scaffold maintained its 3D structure, including vasculature, glomeruli, and tubules.^[^ ^103^ ^]^ Micro‐CT scanning confirmed the integrity, patency, and connectivity of the vascular network. AFM, employing a sharp, fine tip controlled by piezoelectric motors to scan the tissue surface, is frequently utilized to corroborate TEM observations. The deflection of the tip during scanning is monitored by a laser diode detector and transformed into 3D topographical images. Unlike TEM and SEM, AFM measures the inter‐fibrillar gaps and acts as a non‐destructive screening tool for decellularized tissues.^[^ ^104^, ^110^ ^]^

Maurice and colleagues initially employed X‐rays to investigate structural features pertaining to the arrangement of collagen fibers in corneal tissues, enabling the quantification of the orientation and content of collagen fibers across the cornea and limbus, with changes in fiber arrangement being readily detected via X‐ray scattering patterns.^[^ ^94^, ^105^, ^111^ ^]^ Furthermore, this approach obviates the need for tissue processing steps that could potentially compromise tissue architecture. Optical coherence tomography (OCT) and ultra‐high‐resolution OCT, which are non‐invasive imaging techniques, provide micron‐scale resolution and can measure cross‐sectional structural features and the thickness of tissues up to 2 mm. OCT, eliminating the need for fluorescent labeling of tissues and being relatively inexpensive compared to confocal and multiphoton techniques, is well‐suited for scanning decellularized tissues and facilitates non‐destructive, rapid, sterile, in situ, real‐time studies.^[^ ^94^ ^]^ Park and colleagues utilized swept‐source OCT for the in vivo non‐invasive monitoring of rabbit corneas following implantation with 3D bioprinted decellularized collagen sheets.^[^ ^106^ ^]^ Ultra‐high‐resolution OCT enables visualization of intracorneal structural features with an axial resolution of 2 to 3 microns and can distinguish between layers such as Bowman's and the stroma, providing a valuable alternative for rapid tissue characterization following decellularization.^[^ ^112^ ^]^

### 4.2 The multicomponent analysis of dECM scaffold

The principal constituents of decellularized scaffolds comprise ECM molecules, including collagen, elastin, fibronectin, and various growth factors. Analysis of decellularized scaffolds concentrates on key ECM constituents, which are quantifiable and identifiable through biochemical and molecular biology techniques, such as immunohistochemistry, mass spectrometry, PCR, and Western blotting. These bioactive proteins are employed in the fabrication of biomaterials and tissue engineering applications, to regulate cellular functions such as growth, proliferation, and migration.

**FIGURE 4:**
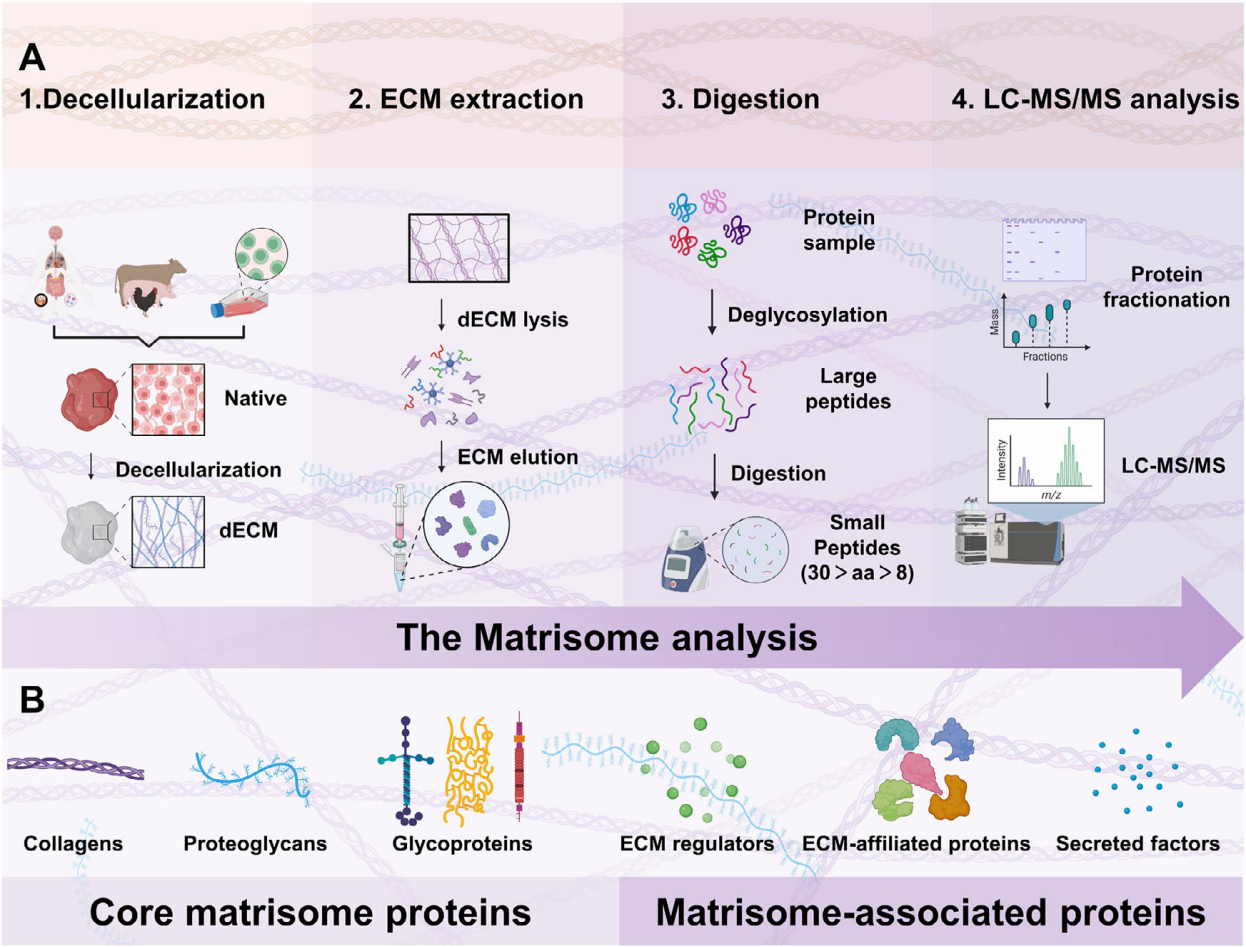
The matrisome analysis workflow diagram. (A) The analysis of matrix composition of dECM consists of four steps: decellularization, extraction of ECM protein, enzymatic hydrolysis and mass spectrometry analysis. (B) The matrisome consists of core matrisome proteins and matrisome‐associated proteins, where core matrisome proteins includes collagens, proteoglycans, glycoproteins, and matrisome‐associated proteins includes ECM regulators, ECM‐affiliated proteins, secreted factors. Created with BioRender.com.

With advancements in proteomics, the characterization of matrix components has become feasible. The field of proteomics that is dedicated to comprehensive ECM analysis is known as matrisomics. Hynes and Naba first presented the matrisomics concept in 2012, establishing a matrisome database (http://matrisomeproject.mit.edu/) which classifies ECM into core matrisome proteins, including collagens, proteoglycans, and glycoproteins, and matrisome‐associated proteins, encompassing secreted factors, regulators, and ECM‐affiliated proteins, thus providing a reliable reference for comprehensive dECM analysis.^[^ ^117^, ^118^ ^]^ Bottom‐up proteomics is now the preferred method for ECM proteome analysis. Decellularization represents an effective technique for ECM protein enrichment, and current matrisome analysis workflows are delineated based on the process depicted in Figure 4.

#### 4.2.1 Collagens

Collagen, the predominant structural protein in the ECM, provides an appropriate 3D microenvironment that promotes cell adhesion, proliferation, and differentiation, thereby facilitating tissue regeneration and repair.^[^ ^119^ ^]^ Therefore, the presence of collagen in decellularized scaffolds is essential, fulfilling pivotal structural and functional roles. Analysis of collagen in decellularized scaffolds entails quantification and assessment of distribution, utilizing methods such as staining, microscopy, and spectroscopy; the Russel‐Movat pentachrome staining kit is utilized for ECM component detection.^[^ ^120^ ^]^ Additionally, the structure and function of collagen are examined through techniques such as the tetrahedral molecular model and biomechanical testing. Multiphoton NIR femtosecond laser scanning microscopy acts as a novel optical tool for non‐confocal 3D imaging of endogenous fluorophores and ECM structures like collagen and elastin.^[^ ^121^, ^122^ ^]^ Collagen displays strong second harmonic generation, whereas elastin demonstrates weaker autofluorescence in the green‐blue spectrum.^[^ ^123^ ^]^ Furthermore, collagen's non‐centrosymmetric triple helix structure and high crystallinity make it more effective in generating second harmonic waves than elastin. Different types of collagen in decellularized scaffolds perform distinct roles in cell differentiation and tumor migration. Wen et al. found that the ECM protein COL4A2 promotes osteogenic differentiation of PDLSCs by negatively regulating the Wnt/β‐catenin pathway, suggesting a potential strategy for bone defect repair.^[^ ^124^ ^]^

#### 4.2.2 Proteoglycans

Proteoglycans, interspersed among collagen fibrils in various ECMs, confer properties beyond mere structural strength. These glycoproteins, with attached GAGs, assume an extended conformation due to their high negative charge, attracting water and divalent cations such as calcium, which imparts space‐filling and lubricating functions. GAGs, particularly heparan sulfate, adhere to numerous secreted factors and growth factors within the ECM. Proteoglycans engage in interactions with collagen, fibronectin, laminin, and elastin in the ECM, contributing to the creation of a matrix with specialized tissue properties. Similar to collagen, the ECM in different tissues incorporates diverse types and quantities of GAGs and proteoglycans, tailored to specific functions. For instance, cartilage and growth plates in long bones exhibit high concentrations of chondroitin sulfate proteoglycans. The hydrophilic nature of chondroitin sulfate enables it to occupy space and confer volume, which is vital for growth plates in epiphyses. Insufficient or deficient sulfation of chondroitin sulfate can diminish growth plate volume, resulting in stunted limb development and deformities. The anionic groups in GAGs attract divalent cations, which are vital for tissue calcification and bone salt deposition. In the cornea, proteoglycans, principally keratan sulfate and dermatan sulfate with high protein content, are essential in maintaining corneal matrix transparency. Various ECM components, including GAGs and proteoglycans, interconnect to form gels with varying pore sizes or charge densities, which integrate the ECM and act as a sieve for molecular and cellular passage, a function particularly critical in the glomerulus and vascular basement membranes.

#### 4.2.3 Glycoproteins

Glycoproteins in the ECM, including elastin, fibronectin, and laminin, play multifaceted roles that are indispensable for tissue structure and function. Elastin, a key ECM protein, imparts elasticity and resilience to tissues. While decellularization can preserve elastin content, the process might also result in reduced quantities and potential damage to the protein, thereby affecting the mechanical properties of scaffolds. Studies have utilized a variety of staining and imaging techniques to evaluate elastin integrity post‐decellularization, underscoring the importance of preserving its quality for scaffold functionality. Fibronectin is implicated in various cellular processes, encompassing migration, proliferation, differentiation, and wound healing. It significantly contributes to the biomechanical strength of scaffolds and promotes cell adhesion, which is crucial for scaffold stability and biocompatibility. Decellularization can degrade fibronectin, particularly its higher molecular weight forms, potentially impacting the activation of pathways such as Wnt/β‐Catenin that are critical for organ function maturation, including in the liver.^[^ ^92^ ^]^ Laminin, predominantly located in the basement membrane, is critical for cell behavior, encompassing growth and differentiation. It enhances cell adhesion and proliferation within scaffolds and has the potential to influence differentiation pathways. For instance, laminin‐5 has been demonstrated to promote mesenchymal stem cell (MSC) proliferation and reduce chondrogenic differentiation, potentially benefiting bone tissue development.^[^ ^125^ ^]^ The enrichment of laminin in decellularized liver matrices has been explored with the aim of creating transplantable grafts.^[^ ^126^ ^]^ The decellularization process can occasionally affect the function of laminin, underscoring the need for further research to optimize methods that preserve its beneficial properties within scaffolds. The preservation of laminin in decellularized scaffolds from diverse organs demonstrates its significance in supporting cell adhesion and its potential in advancing tissue engineering applications.^[^ ^127^ ^]^ These glycoproteins, together with the other components of the ECM, constitute a complex network that underpins cellular functions and tissue integrity. The challenge in decellularization resides in effectively removing cellular material while retaining the structural, biochemical, and biomechanical cues provided by these proteins to engineer functional scaffolds for regenerative medicine.

#### 4.2.4 ECM‐bound growth and secreted factors

Numerous growth factors that bind to ECM proteins are considered integral components of the ECM. It is widely acknowledged that growth and secreted factors bind to GAGs,^[^ ^128^ ^]^ particularly heparan sulfate. However, growth factors do bind to specific structural domains of ECM proteins in particular cases. For instance, fibronectin binds to various growth factors, such as VEGF, HGF, PDGF, and is recognized for its binding to BMPs through VWC/chordin and follistatin domains found in numerous ECM proteins.^[^ ^129^ ^]^ Transforming growth factor (TGF) specifically interacts with the TB domain in latent TGF‐β binding protein (LTBP), which subsequently associates with matrices rich in fibrillin and fibronectin. The ECM can serve as a reservoir or sink for these factors, with numerous examples including chemotactic factors and pivotal developmental signals such as VEGF, Wnts, Hedgehog proteins (Hhs), BMPs, and FGFs. These factors establish gradients that regulate developmental patterning, with some gradients notably influenced by ECM binding.

### 4.3 Decellularized scaffolds and cell interactions

ECM scaffolds offer a three‐dimensional structure and signaling platform that influence the biological behavior of cells including adhesion, migration, proliferation, and differentiation. These characteristics contribute to the development of dECM as biomaterials in biological and medical applications.

Regarding cell adhesion, proteins and peptides within ECM scaffolds facilitate the attachment of cells to the scaffolds by binding to integrin receptors, which serve as biosensors for intracellular and extracellular signals on the cell surface. Such adhesion can offer the mechanical support necessary for promoting the stable localization and function of cells. Moser et al. discovered that alin and kindlins proteins regulate the activity of integrins in transmitting signals from the cell interior to the cell exterior. In addition, external signals are transmitted to the interior of the cell through the interaction of integrins and their ligands. Collagen, laminin, fibritin and RGD in ECM scaffolds can interact with 11 integrins.^[^ ^130^ ^]^ These interactions ensure adhesion and the transfer of information between cells and the ECM. For instance, interactions between integrins and the ECM are implicated in regulating axon growth and pathfinding.

ECM scaffolds can modulate cell proliferation and differentiation by offering both mechanical support and biochemical signals. Various bioactive molecules within ECM scaffolds, including ECM proteins, growth factors, and cell adhesion proteins, can direct the regulation of cell proliferation and differentiation. For example, fibroblast proliferation is accelerated on fibronectin matrices but decelerated on laminin matrices. Conversely, the proliferative response of epithelial cells to fibronectin and laminin matrices exhibited an opposite trend. Furthermore, ECM components such as laminin, fibronectin, and collagen can enhance Schwann cell proliferation in vitro and increase the number of neurites per cell, illustrating their ability to stimulate Schwann cells to release factors promoting neurite growth.^[^ ^131^ ^]^ Regarding cell differentiation, research has revealed that certain matrix‐binding proteins serve as markers for stem cell surface proteins. For example, CD49a‐f is expressed on the surface of basal epithelial progenitor cells and tumor stem cells. CD29 is highly expressed in neural stem cells but diminishes upon their differentiation into neurons.^[^ ^132^ ^]^ Simultaneously, ECM can also furnish a microenvironment for stem cell differentiation. For example, laminin constitutes the in vivo ECM niche of trophoblastic stem cells (TSCs), with collagen and laminin serving as the ligands of the TSC niche.^[^ ^133^ ^]^ Laminin‐425 constitutes a major component of bone marrow ECM and also influences the circulation and homing of hematopoietic stem/progenitor cells within the bone marrow.^[^ ^134^ ^]^ During chondrogenesis, fibronectin interacts with other ECM components to direct cell differentiation.^[^ ^135^ ^]^ Furthermore, the depletion of tissue inhibitors of metalloproteinases (TIMP) proteins diminishes the germ cell microenvironment's capacity to generate new cysts, thereby jeopardizing the normal organization and function of the adult female germline stem cell microenvironment. ECM participates in the differentiation of tendon stem cells (TSPCs) and the fate determination of epidermal stem cells.^[^ ^136^, ^137^ ^]^ Interestingly, tendon‐derived ECM promotes the tendon phenotype of TSPCs and suppresses osteogenic differentiation by modulating tendon—and bone‐specific transcription factors Scleraxis and Runx2. However, bone‐derived dECM induces osteogenic differentiation of TSPCs.^[^ ^137^ ^]^

## 5 POSTPROCESSING OF DECM SCAFFOLDS FOR APPLICATION

Postprocessing of dECM scaffolds involves a series of steps performed after decellularization. Sterilization and preservation of dECM scaffolds are critical considerations as biomedical materials. Numerous graft forms can be fabricated from decellularized tissues, mirroring the diversity of decellularization methods. Common forms include “2D” scaffolds, ECM powders, hydrogels, composite grafts, and whole organs. Physical and chemical properties vary among graft types, with the intended application guiding the selection of graft form. Simpler graft structures, such as skin and vascular grafts, have achieved clinical success, while more complex transplants like whole organs continue to present challenges.

### 5.1 Approaches to decellularized scaffold processing: crosslinking and functional modification

The degradation and metabolism of decellularized scaffolds are critical factors limiting the speed of progress in tissue engineering. Indeed, the degradation and metabolism of decellularized scaffolds are complex processes influenced by various factors. Currently, scaffold‐cell and scaffold‐tissue interactions are primarily influenced by surface modification or functionalization, thereby impacting the degradation and metabolic processes of scaffolds.

Gao et al. discovered that EDAC serves as an effective cross‐linking agent for acellular meniscus scaffolds. Moreover, the cross‐linking process effectively enhances the anti‐degradation and mechanical properties of scaffolds due to the connection and fusion between collagen fibers and tissue blocks.^[^ ^138^ ^]^ Interestingly, once autologous skin is regenerated, the degradation products of dECM have little toxicity and even have the ability to induce tubular structure formation and skin regeneration.^[^ ^139^ ^]^ The degradation rate of dECM scaffolds should be consistent with the process of bone reconstruction, which affects the time of cell growth, and improper speed may lead to transplantation failure.^[^ ^139^, ^140^ ^]^ For therapeutic applications, these polymers are typically processed into porous scaffolds, hydrogels, particles, or films implanted in the body, where they are typically degraded by enzymes into non‐toxic end products. Synthetic polymers are primarily degraded via simple chemical hydrolysis into non‐toxic byproducts, which are eliminated through normal metabolic pathways.^[^ ^141^ ^]^ However, the enzymatic degradation of decellularized scaffolds is not as easily predictable and controllable as chemical hydrolysis due to the absence of dependence on local enzyme concentration. Although the degradation kinetics of these biomaterials may not be easy to control or predict, they are still effective if local, short‐term reactivity effects are sufficient. At present, some ECM components in decellularized scaffolds alter the biocompatibility and degradation performance of tissue‐engineered biomaterials through specific treatments (crosslinking or modification). For example, collagen can be cross‐linked through specific chemical glycoylation procedures or heat treatment to give tissue engineered materials tailored mechanical properties, degradation properties, and water absorption properties. Injectable hydrogels supplemented with specially modified elastin are more adaptable in terms of gelation and biodegradation, adjustable porosity and pore size, and exhibit good mechanical properties and/or structural stability.^[^ ^142^, ^143^, ^144^ ^]^ In addition, SIS is cross‐linked in some applications to reduce its blood compatibility, regulate the mechanical properties of scaffolds, and degrade rates. Once the scaffold material degrades into macromolecules, it is released at the target site as a signal for cell indicators, offering a biomedical approach to fine‐tune the cellular environment in vivo.^[^ ^145^ ^]^

#### 5.1.1 Crosslinking

Decellularization can compromise the biomechanical properties of the ECM, necessitating the use of physical and chemical crosslinking techniques to preserve the 3D structure of the dECM and enhance the mechanical strength of scaffolds. Chemical crosslinking commonly utilizes agents such as aldehydes (e.g., formaldehyde, glutaraldehyde), epoxides, and isocyanates to link molecular structures within the scaffold, thus enhancing stability and mechanical integrity. Glutaraldehyde is frequently used as a crosslinker for collagen‐based materials, applied in the perfusion crosslinking of decellularized renal scaffolds; nevertheless, its cytotoxicity may impede subsequent research. Conversely, dialdehydes and genipin are employed to mitigate cytotoxicity. Importantly, research has shown that crosslinking can promote cell adhesion and differentiation. For example, the application of EDC crosslinking combined with chemical extraction for tissue decellularization has demonstrated efficacy in enhancing mesenchymal stem cell adhesion and differentiation. However, chemical crosslinkers may compromise scaffold biocompatibility and potentially induce toxicity.^[^ ^146^ ^]^ Furthermore, the complete removal of crosslinking agents presents challenges and poses potential risks.

#### 5.1.2 Modification

To enhance porosity and bolster cell infiltration, surface modification techniques such as laser sintering, solvent casting, particulate leaching, and electrospinning have been refined. Laser sintering utilizes a laser beam to selectively sinter the material's surface, resulting in a high density of micro‐pores, which effectively facilitates decellularization by generating micropores on tissue surfaces. Specifically, in cartilage, laser modification has been shown to promote migration and dedifferentiation of reseeded chondrocytes while simultaneously preserving the mechanical strength of the native tissue. Solvent casting and particulate leaching, methods in which a uniform salt solvent evaporates, producing a sponge‐like porous structure in water, are recognized as optimal templates for cell permeation and nutrient transfer. Moreover, inorganic polymer coatings and peptide modifications represent recent innovations in ECM modification. Researchers have strived to covalently attach bioactive peptides to the inner surface of dECM, leading to improved cellular adhesion, proliferation, and differentiation. Additionally, ECM‐derived peptides featuring signaling domains (ECM fragments) have been employed to augment other biomaterials, enhancing functionality via interaction with cell membrane receptors.

### 5.2 Diverse application forms of decellularized scaffolds

**FIGURE 5:**
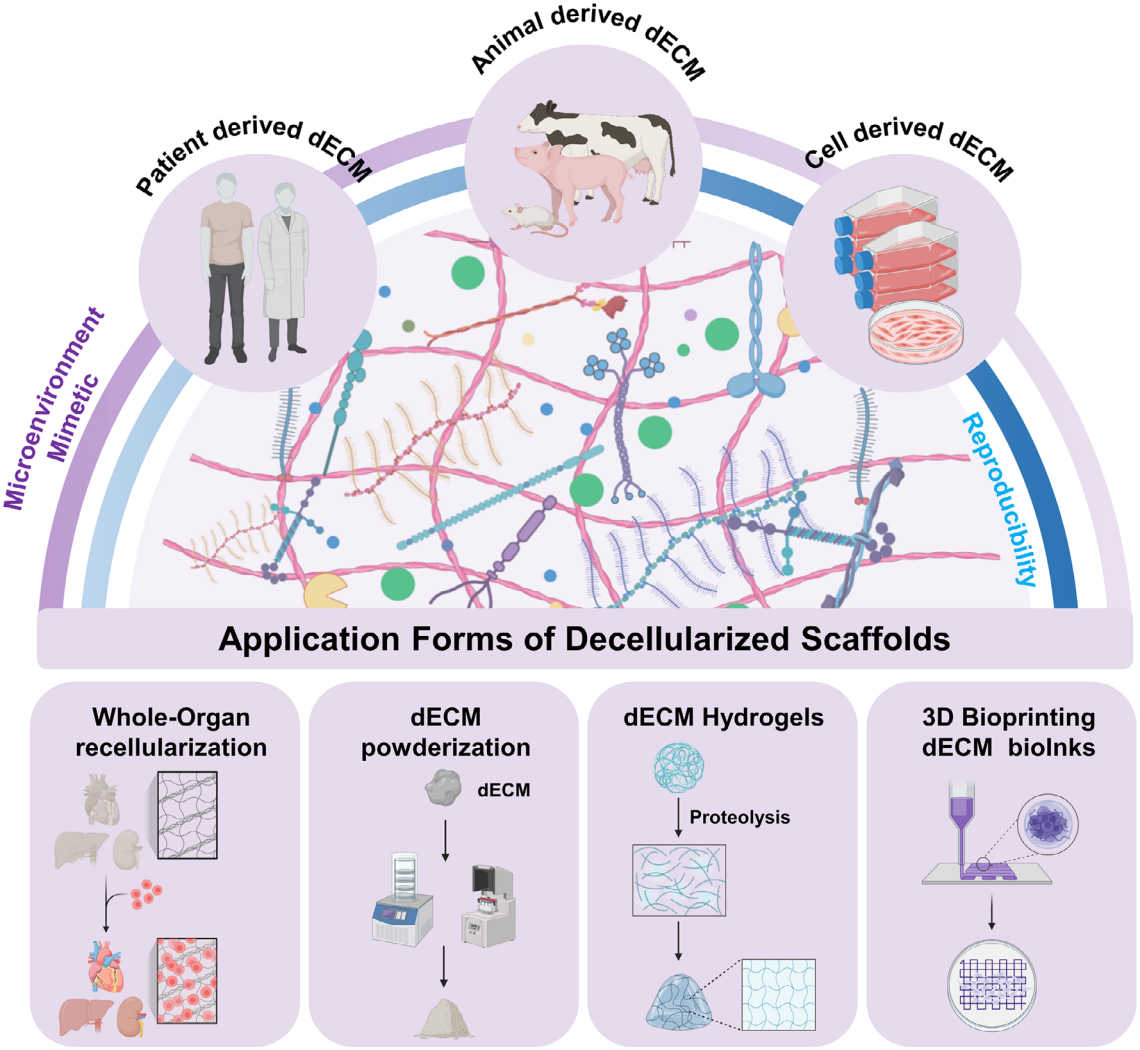
Diverse application forms of decellularized scaffolds. The application forms of decellularized scaffolds mainly include four aspects: whole‐organ level recellularization, powderization, hydrogels fabrication and 3D bioprinting inks. Created with BioRender.com.

To advance the clinical application of decellularized scaffolds, various transplantable forms have been developed, including ECM powders, hydrogels, and whole organs (Figure 5). These forms vary in their physical and chemical properties, with the choice of processing form determined by the intended application.

#### 5.2.1 Applications at the whole‐organ level

In clinical practice, decellularized whole tissues are preferred for transplantation due to their retained structure, unlike derivatives like powders or hydrogels. Widely used decellularized dermal products—GraftJacket, Integra, Dermagraft, Apligraft, and Allopatch—primarily composed of collagen, serve as versatile skin grafts for tendon, bone, hernia repairs, and wound healing.^[^ ^147^ ^]^ Recellularization, the reintroduction of cells into ECM scaffolds to form functional tissues, has been exemplified by Ott's group, which created contractile 3D cardiac tissues from decellularized matrices and iPSC‐derived cardiomyocytes, offering potential for heart repair.^[^ ^148^ ^]^ Similarly, Brouki Milan's work with decellularized dermal matrices seeded with mesenchymal stem cells from human umbilical cords has shown promise in enhancing diabetic wound healing.^[^ ^149^ ^]^ Recellularization outcomes hinge on cell type, technique, density, and culture conditions. Despite its potential, this approach is limited by risks such as cell contamination, graft durability, disease transmission, and vascular blockages.

#### 5.2.2 Powderization of decellularized scaffolds

Decellularized ECM powders, produced through freeze‐drying and pulverizing tissues, offer unmatched versatility in tissue engineering not observed with whole tissues. These powders can be customized to fill irregular defects and facilitate minimally invasive implantation. Key parameters including particle size, solubility, and crosslinking are meticulously regulated during powder production. ECM powders have been utilized to create ECM “papers” by transforming powdered suspensions into inks for controlled casting and drying, thereby allowing for precise shape customization. Such “papers” have been demonstrated to support adhesion, viability, and proliferation of human MSCs across diverse tissues.^[^ ^150^ ^]^ Our research entails co‐culturing liver stem cells with decellularized liver ECM powders to form micro‐liver spheroid organoids, thereby replicating the liver's microenvironment and enhancing hepatocyte function. This platform enhances metabolic activity, increases sensitivity to hepatotoxic substances, and facilitates high‐throughput analysis of drug effects and liver toxicity pathways.^[^ ^151^ ^]^

#### 5.2.3 Fabrication of hydrogels using decellularized scaffolds

DECM powders can be transformed into hydrogels, making them suitable for injectable and 3D printable biomatrices and thereby expanding their applications. These hydrogels form three‐dimensional networks capable of swelling, retaining water, and mimicking the flexibility of natural tissues, all the while preserving tissue‐specific biochemical cues.^[^ ^152^ ^]^ Hydrogel formation entails the pepsin solubilization of dECM, thereby enabling the self‐assembly of collagen fibers into a 3D network. For instance, methacrylated bone ECM hydrogels, synthesized from demineralized and decellularized human bone, exhibit tunable mechanical properties and facilitate vascular network formation due to the angiogenic molecules present within the bone ECM.^[^ ^153^ ^]^ Similarly, incorporating PEGDA into DAFM hydrogels strengthens them without compromising porosity, and TGF‐β1‐enriched hydrogels have demonstrated promise in intervertebral disc repair in vivo.^[^ ^154^ ^]^ dECM hydrogels are being investigated for the repair of a variety of tissues, including cardiac and liver tissues, and for conditions such as Type 1 diabetes, peripheral artery disease, and skin wounds. Crosslinking methods significantly influence the hydrogels' properties and effectiveness. Comparative studies have scrutinized various crosslinking techniques, evaluating mechanical strength, degradation resistance, and biocompatibility, all of which are crucial for successful tissue repair and regeneration.^[^ ^155^ ^]^ These findings highlight the potential of dECM hydrogels, modified through various crosslinking methods, to advance regenerative medicine and tissue engineering.

#### 5.2.4 Development of 3D bioprinting inks derived from decellularized scaffolds

3D bioprinting, a technique for creating tissue‐like structures, utilizes bioinks for the precise placement of cells in three dimensions. dECM‐based bioinks, mimicking tissue‐specific microenvironments, are more effective than homogeneous bioinks but frequently suffer from low viscosity due to ECM protein loss during decellularization, impeding bioprinting performance.^[^ ^98^ ^]^ To address this issue, researchers have incorporated biomaterial‐based additives such as alginate, silk proteins, and gelatin into dECM bioinks, thus enhancing printing conditions and cell viability.^[^ ^156^ ^]^ The bioprinting process also depends on the crosslinking of materials to transition from sol to gel phases. Traditional thermal crosslinking, although it preserves cell activity, lacks the mechanical robustness and rapid gelation necessary for constructing large 3D structures. Photocrosslinking, employing photoinitiators and photosensitizers, presents a novel solution for strengthening dECM gels, facilitating layer‐by‐layer solidification during or after printing.^[^ ^157^ ^]^ Renal dECM methacrylated for photocrosslinking has been utilized to print functional kidney microtissues, fostering cell maturation in a kidney‐specific microenvironment. Similarly, methacrylated dECM from porcine skeletal muscle, when combined with sacrificial fibrillated PVA, has been bioprinted into aligned skeletal muscle structures. This process ensures myoblast alignment and differentiation, crucial for myotube formation, by employing UV light for crosslinking and removing PVA post‐printing.^[^ ^158^ ^]^ This technique capitalizes on the dECM's biochemical and topographical cues for myogenic development, showcasing the potential of dECM‐based bioinks for tissue engineering applications.

## 6 CONTEMPORARY UTILIZATION OF DECELLULARIZED SCAFFOLDS IN TISSUE ENGINEERING

The development of dECM‐based scaffold technology has markedly progressed, and is now more frequently considered for strategies in tissue engineering and regenerative medicine. Additionally, dECM has demonstrated considerable potential in constructing in vitro physiological and disease models.

### 6.1 Decellularized scaffolds: Addressing the transplant donor shortage dilemma

The use of decellularized scaffolds prevents immunogenic rejection in clinical transplantations and maintains structures and components analogous to native organs. Currently, they are employed in whole‐organ engineering to address the shortage of transplant donors, involving the reseeding of patient cells into decellularized tissues (recellularization) for transplantation, although this remains at the preclinical stage. On the other hand, decellularized tissue‐based products, like hydrogels for 3D bioprinting of organs or tissues, hold potential for regenerative purposes, potentially alleviating donor scarcity. Moreover, the implantation of decellularized tissues in vivo can facilitate post‐injury regeneration and wound healing.

#### 6.1.1 Engineered tissue constructs for transplantation and regenerative applications

**FIGURE 6:**
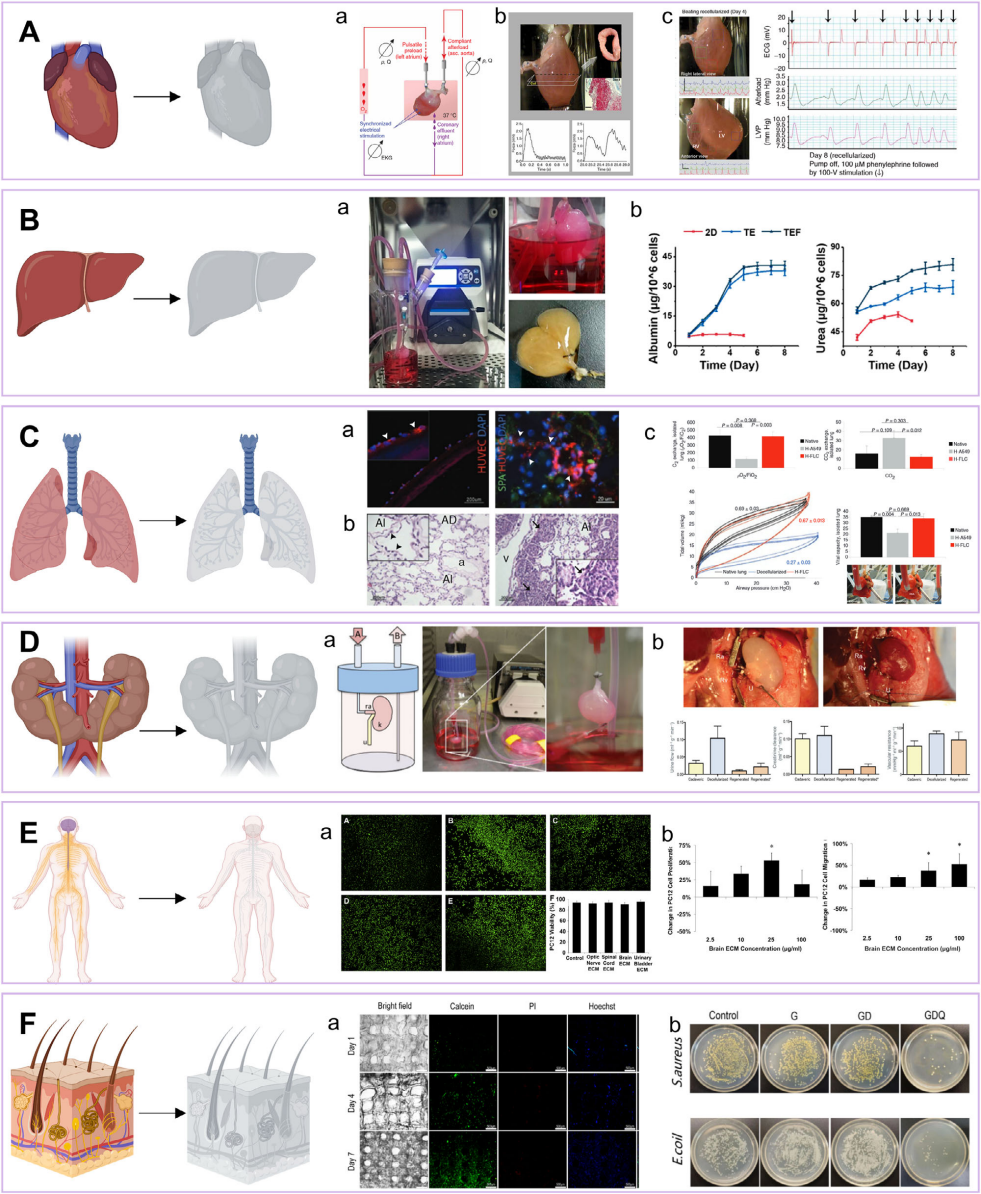
Applications of decellularized scaffolds in tissue engineering. (A) The decellularized heart scaffold was recellulated to form (a,b) a working perfusion bioartificial heart‐like structure, which showed (c) electrical response and contractile function. Reproduced with permission.^[^ ^148^ ^]^ Copyright 2008, Springer Nature. (B) After recellularization, the decellularized liver matrix produces transplantable liver grafts to support liver‐specific functions, including albumin secretion and urea synthesis. Reproduced with permission.^[^ ^189^ ^]^ Copyright 2018, American Chemical Society. (a) Photograph of circulating perfusion system and the TE liver after recellularization with ≈30 million hepatocytes. (b) Analysis of albumin secretion and urea synthesis of HepG2 cells under 2D, TE, and TEF liver cultures. (C) The decellularized lung scaffold performs gas exchange function after implantation by epithelial cells and endothelial cells. Reproduced with permission.^[^ ^175^ ^]^ Copyright 2010, Springer Nature. (a) Fluorescence micrographs of reendothelialized lung constructs (scale bars, 200 µm). (b) H&E‐stained low‐ (×10) and high‐power views (×40, insets) of A549‐seeded lung constructs (scale bars, 200 µm). (c) Blood gas analyses of pulmonary venous effluent, the dynamic pressure/volume relationship during five respiratory cycles and vital capacity of native lung, HUVEC‐ and A549‐seeded lung (H‐A549), and HUVEC‐ and fetal lung cell–seeded lung (H‐FLC). (D) By the recellularization of epithelial cells and endothelial cells, the decellularized scaffolds of rat kidney were transformed into bioengineered kidney grafts with the function of urine production after orthotopic transplantation. Reproduced with permission.^[^ ^171^ ^]^ Copyright 2013, Springer Nature. (a) Schematic of cell seeding and whole organ culture of decellularized rat kidneys. (b) Average urine flow rate (mL min^−1^), average creatinine clearance and vascular resistance for decellularized, cadaveric, and regenerated kidneys perfused at 80 mmHg and regenerated kidneys perfused at 120 mmHg (regenerated*). (E) The bioscaffolds derived from the central nervous system have cytocompatibility after co‐culture with PC12 cell lines *in* vitro, and stimulate proliferation, migration and differentiation, thus contributing to functional recovery after CNS injury. Reproduced with permission.^[^ ^177^ ^]^ Copyright 2012, Elsevier. (a) Cytocompatibility of CNS ECM scaffolds. Magnification is 400×. (b) Mitogenic effects and chemotactic effects of CNS ECM scaffolds. (F) The Gel‐dECM‐Qcs (GDQ) composite scaffold formed by 3D printing technology combining decellularized pig skin scaffold, temperature sensitive gelatin (Gel) and quaternized chitosan has good mechanical properties, biocompatibility and antibacterial ability. Reproduced with permission.^[^ ^182^ ^]^ Copyright 2023, Elsevier. (a) Live/dead staining of L929 cells in different pure/composite scaffolds including Gel, Gel‐dECM and Gel‐dECM‐Qcs. Scale bar = 500 µm. (b) Representative images of colony count of the scaffolds against *Escherichia coli* and *Staphylococcus aureus*. Organ schematic images from BioRender.com.

Organ transplantation remains the principal treatment for end‐stage organ failure, yet many patients succumb while awaiting transplants owing to donor scarcity. Tissue engineering and regenerative medicine provide innovative solutions to this shortage. Decellularized scaffolds preserve the structure and composition of native tissues along with specific bioactive factors, creating a unique microenvironment for cell adhesion, proliferation, and differentiation. Seeding these scaffolds with cells can lead to tissue regeneration, thereby addressing the critical issue of organ and tissue donor insufficiency. Table 3 and Figure 6 provide an overview of current regenerative strategies based on decellularized scaffolds.

**TABLE 3:** The application of dECM in tissue engineering.

Application	Source	Function	Reference
Adipose tissue	Human	Enhance cellularity and angiogenesis, support cellular infiltration, differentiation, construct in vitro bionic systems	[159, 160]
Bladder	Porcine	Promote smooth muscle, neurons and blood vessels regeneration	[161]
Blood vessel	Human (umbilical arteries)	Promote smooth muscle cells adhesion, proliferation, and migration	[60]
Cartilage	Porcine (articular cartilage) Human (amniotic membrane)	An amniotic membrane with PDLSCs/osteoblasts has therapeutic potential for bone defects	[162]
Cornea	Human, porcine	An excellent recellularization capacity in vitro, corneal transplant, treat retina damage	[163, 164]
Dental pulp	Porcine	Pulp regeneration	[165]
Esophagus	Human, porcine	Artificial esophagus, promote esophageal tissue remodeling	[166, 167]
Heart	Rat	Engineer a bioartificial heart, enhance maturation of cardiomyocytes	[148, 168]
Heart valve	Porcine (aortic valve)	Support recellularization in tissue‐engineered heart valve, treatment of heart valve disease	[169, 170]
Kidney	Porcine, rat	Support kidney functions such as urine production, guide cell attachment and growth, biocompatibility and hemocompatibility	[171, 172]
Liver	Human, porcine	Help cellular proliferation, drug hepatotoxicity evaluations, develop the transplantable recellularized liver graft	[126, 151, 173]
Lung	Rat, mouse	Generate functional pulmonary tissue, lung transplant	[174, 175, 176]
Nerve	Rat (peripheral nerve) Porcine (peripheral nerve)	Maintain basal lamina integrity and flexibility, support Schwann cell proliferation and preserve cell morphology, promote the activation of M2 macrophages associated with a constructive remodeling response	[177, 178, 179]
Ovary	Human	Construct bioengineered ovaries	[180]
Pancreas	Human	Beta cell replacement medicine	[181]
Skin	Human, fetal bovine	Good biocompatibility, promote tissue engineered composite scaffolds, accelerate of skin regeneration	[182, 183, 184]
Small intestinal submucosa	Porcine	Repair various tissues and organs	[61]
Tendon	Porcine	The substitute for tendon repair, decellularized tendon slices	[185, 186]
Trachea	Rabbit	Improve the adherence rate of cells with perfect cell biocompatibility, an ideal scaffold for trachea tissue engineering	[187, 188]

Ott et al. successfully optimized the decellularization of rat hearts, creating scaffolds with preserved vasculature, valves, and chambers.^[^ ^148^ ^]^ Upon repopulation with cardiomyocytes or endothelial cells and subsequent culture, these constructs regain cardiac function. dECM scaffolds have demonstrated promise in myocardial infarction therapy by mitigating adverse remodeling and promoting cardiac repair. They facilitate vascular and cardiomyocyte regeneration as well as ECM remodeling. Initiatives to develop decellularized heart valves with anti‐calcification properties have been undertaken, with hypoxic bioreactor conditioning demonstrating increased cellular infiltration in valve leaflets.^[^ ^169^, ^170^ ^]^ Cardiac dECM patches have also been engineered for myocardial repair, with studies indicating that bone marrow cells seeded on these patches exhibit robust viability and differentiation.^[^ ^190^ ^]^ Jang et al. developed a dECM‐based bioink for 3D printing cell‐laden cardiac patches, which demonstrate potential for vasculogenesis and cardiac regeneration with extended culture.^[^ ^168^ ^]^

dECM scaffolds have emerged as a pivotal innovation in liver tissue engineering, preserving liver‐specific ECM, biliary structures, and vascular networks. Uygun's groundbreaking work in 2010 yielded transplantable grafts from decellularized liver matrices that supported liver functions, including albumin secretion, urea synthesis, and cytochrome P450 activity, and enhanced hepatocyte survival and function post‐transplant.^[^ ^126^ ^]^ Subsequent advancements have concentrated on optimizing decellularization to minimize processing time and preserve vascular integrity. Wang et al. introduced a ‘four‐step' decellularization technique utilizing SDC and PLA2, producing scaffolds in just 40 min that facilitated rapid maturation of liver stem cells into functional hepatocytes with extended viability. These dECM scaffolds show considerable promise for treating acute and chronic liver conditions.^[^ ^173^ ^]^

Ott's seminal 2010 work in bioartificial lung development via decellularization preserved the lung's vascular system, airways, alveoli, and ECM proteins, while also maintaining alveolar septa and surface area. By seeding epithelial and endothelial cells onto the decellularized scaffold and perfusing it within a bioreactor to simulate lung physiology, they created a lung construct capable of gas exchange, comparable to native lungs, and sustaining in vivo function for 6 hours post‐transplant.^[^ ^175^ ^]^ Although this work demonstrated the feasibility of bioartificial lung engineering, further research is required to enhance graft regeneration and function. Recent advancements by Young et al. have focused on enhancing epithelial and alveolar barriers by augmenting dECM with basement membrane proteins such as laminin or fibronectin, resulting in improved barrier function in vitro. This was correlated with an increased expression of adhesion proteins, notably Claudin‐18, which may contribute to alveolar stability.^[^ ^176^ ^]^ Decellularized lung matrices, therefore, offer promising avenues for the treatment of pulmonary disorders such as chronic obstructive pulmonary disease. Similar to lung decellularization, kidney decellularization is typically conducted via the vascular system, utilizing the renal artery and ureter. In 2009, Ross et al. achieved the first decellularization of an entire rat kidney through arterial perfusion with detergents and enzymes such as Triton X‐100, SDS, SDC, and DNase.^[^ ^174^ ^]^ In 2013, Song et al. conducted the first experimental transplantation of a bioengineered kidney in a rodent model, creating decellularized whole‐organ scaffolds that preserved vasculature, glomeruli, and tubules. Once seeded with renal cells and cultured in a bioreactor, the engineered grafts processed waste, reabsorbed electrolytes, produced concentrated urine in vitro, and successfully managed urine production post‐transplantation in vivo.^[^ ^171^ ^]^ Current research endeavors to refine decellularization to maintain vascular and structural integrity, thereby enhancing the prospects for re‐endothelialization, efficient waste filtration, and functional kidney regeneration. dECM scaffolds present a promising solution for the therapy of chronic kidney disease and end‐stage renal disease.

Decellularized bone‐derived scaffolds offer an innovative alternative to traditional bone grafting, harnessing the inherent structural and biochemical cues of bone to facilitate repair and regeneration. These scaffolds preserve the macro‐ and microstructural characteristics of bone, encompassing geometry, porosity, and surface roughness. The rigid matrix of dECM scaffolds replicates the natural collagen structure found in osteoid, known to promote osteogenic differentiation in stem cells adhering to such rigid substrates. The presence of immunomodulatory cytokines such as transforming growth factor‐β (TGF‐β), bone morphogenetic proteins (BMPs), and basic fibroblast growth factor (bFGF) within the dECM scaffolds plays a crucial role in modulating inflammation and supporting the healing process. Furthermore, trace elements such as magnesium and strontium, essential for bone integrity and cellular function, are preserved in the dECM, further contributing to the scaffold's effectiveness in bone repair. To date, dECM bone scaffolds have primarily been sourced from livestock, with studies demonstrating their osteoinductive properties, capable of inducing osteogenic differentiation and bone formation both in vitro and in vivo. When combined with collagen, BMPs, and other growth factors, these scaffolds exhibit enhanced osteogenesis and bone formation capabilities. Enhancing dECM scaffolds with collagen/hydroxyapatite (HA) mixtures and stromal cell‐derived factor 1 alpha (SDF‐1α) not only amplifies the osteogenic potential of MSCs in vitro but also attracts endogenous stem cells upon subcutaneous implantation. Incorporating osteoconductive inorganic materials, such as nano‐hydroxyapatite (nHA), into the dECM scaffolds offers additional cues that further support the adhesion, proliferation, and differentiation of osteogenic cells, rendering these scaffolds highly promising for bone tissue engineering applications.

dECM has also been utilized in the repair of skeletal muscle, particularly in cases of irreversible volumetric muscle loss. Choi et al. fabricated a sinusoidal polystyrene surface coated with decellularized muscle ECM, a combination that facilitated the formation of multinucleated myotubes. Compared to collagen‐coated and uncoated substrates, this configuration yielded well‐aligned myotubes with enhanced myogenic differentiation.^[^ ^191^ ^]^ Subsequent studies have used exogenous growth factors to increase cell recruitment and infiltration, contributing to functional muscle regeneration. Shapiro and colleagues developed a biofunctional scaffold system composed of decellularized ECM and IGF‐1. In vitro tests showed that these scaffolds facilitated greater cell infiltration and elevated expression of myosin heavy chain and myotube formation in C2C12 cells compared to controls. Moreover, these decellularized scaffold systems were evaluated in a rabbit anterolateral tibial muscle defect model, showing greater host cell infiltration and increased myofiber formation compared to collagen and dECM groups.^[^ ^192^ ^]^

In the field of neural injury repair, Crapo applied decellularization techniques to various central nervous system (CNS) tissues, such as optic nerves, spinal cords, and brains. The results indicated that the thoroughly decellularized scaffolds preserved bioactive molecules, including myelin, laminin, neuroinductive bFGF, and the neurotrophic protein nerve growth factor. The resulting scaffolds were demonstrated to regulate the behavior of neuron‐like cells (PC12), including their proliferation, migration, and neural differentiation.^[^ ^177^ ^]^ Moreover, observations revealed that CNS ECM, unlike non‐CNS ECM, exhibited tissue‐specific functionality in PC12 cells. Additionally, dECM can be formulated into hydrogels that, upon minimally invasive delivery, are capable of enhancing astrocyte/axon interactions through direct integration with the host tissue.

dECM scaffolds for repair and regeneration of skin tissue are some of the most extensively utilized applications owing to their preservation of the porous dermal bilayer structure, vascular architecture, adhesion, and elasticity as physical cues, along with bioactive molecules including endogenous growth factors. The preservation of an intact basement membrane region is recognized to facilitate keratinocyte adhesion, and the retention of papillary and reticular dermis structures is known to promote angiogenic cell growth. To date, diabetic foot ulcers (DFUs)—representing a full‐thickness skin deficit—are the subject of active study and treatment using dECM scaffolds.^[^ ^182^ ^]^ Angiogenesis, critical for the delivery of oxygen and nutrients to the wound site, continues to be one of the primary challenges in DFU treatment. dECM scaffolds that exhibit high neovascularization potential, preserving the vascular system and endogenous angiogenic factors, are utilized for DFUs. Research suggests that dECM‐based scaffolds may accelerate wound healing in DFUs by influencing granulation tissue formation, epithelial regeneration, and pro‐angiogenic activity, and could potentially reduce scar formation by shortening the inflammatory phase. Several decellularized products, including AlloDerm Regenerative Tissue Matrix and Oasis Wound Matrix, have been successfully introduced to clinical use. However, the reconstruction of accessory structures like hair follicles and sweat glands, which are essential components for skin formation, remains elusive in clinical products.

#### 6.1.2 Vascularization challenges in tissue‐engineered organs

The vascularization of decellularized scaffolds continues to be a formidable challenge for tissue‐engineered organs. Incomplete re‐endothelialization of the vascular system can result in exposed collagen, the activation of the coagulation system, and subsequent thrombosis, which may culminate in organ blockage, hypoxia, and dysfunction. Consequently, vascularization represents a critical bottleneck for the success and functional performance of complex organ constructs. For instance, rat liver scaffolds re‐cellularized with autologous cells demonstrated a survival time of merely 8 h post‐transplantation in recipients, with anticoagulants extending graft survival to 24 h. Modifying lumens with CD31 antibodies to enhance endothelial cell adhesion was associated with increased recipient pig survival to 24 h. The inclusion of angiogenic factors such as VEGF and bFGF during re‐cellularization markedly enhanced the endothelialization of exposed lumens, with re‐cellularized rat lung tissues capable of engaging in gas exchange for 45–120 min and surviving for up to 3 days post‐transplantation. Furthermore, in response to the shortage of pancreatic islet donors and the challenge of post‐transplant vascular supply, Citro et al. devised a novel method for biomanufacturing functional, vascularized pancreatic organs by implanting islet cells into lung decellularized scaffolds. After 7 days of culture, the islets were anatomically and functionally integrated within the surrounding bioengineered vascular system, yielding a functional, perfusable endocrine organ.^[^ ^193^ ^]^ These studies suggest that utilizing appropriate strategies to effectively enhance endothelialization of scaffold lumens may promote revascularization of tissue‐engineered tissues and thereby improve graft and recipient survival.

### 6.2 Decellularized scaffolds provide a new platform for the establishment of in vitro models

In addition to addressing the shortage of organ donors, scientists are actively exploring other applications of decellularized biomaterials, such as constructing biocompatible delivery systems for cells, drugs, and other therapeutic agents. Various biomaterial scaffolds have the potential for use in in vitro model research. Because of their unique advantages, natural decellularized scaffolds are particularly suitable for developing 2D or 3D in vitro models for cell culture, studying healthy or pathological tissues, and providing a robust platform for drug screening.

#### 6.2.1 Decellularized scaffolds provide a biomimetic, tissue‐specific microenvironment for cell and organoid culture

DECM hydrogels provide a biomimetic environment conducive to cell growth, rendering them a valuable tool for direct human cell culture and the derivation of organoids. These advancements have expanded the clinical potential of human organoids by offering a scaffold that retains the ECM's architecture and biochemistry despite the absence of the original cells, thus establishing an optimal environment for new cell implantation and proliferation. The efficacy of these dECM hydrogels in facilitating cell and organoid culture depends on several factors, including the selection of suitable cell types, refinement of cell seeding techniques, and provision of favorable culture conditions. Interactions at the molecular and cellular levels—between the cells and the scaffold, among the cells, and between the cells and their microenvironment—are critical for the effective growth of cells and development of organoids. Brancato et al. showcased the potential of dECM scaffolds by introducing various cancer cell types into decellularized sheep skin. The scaffold not only preserved the original tissue structure but also offered a biomimetic environment conducive to cancer cell adhesion and proliferation. This interaction permits the modulation of cell behaviors, including proliferation, migration, and differentiation, contingent on the tissue's condition and the represented pathological or physiological state, thus offering a cost‐effective alternative to traditional porous scaffolds composed of synthetic biomaterials. Ganjibakhsh et al. employed a decellularized amnion membrane (DAM) to establish a 3D cell culture model for the induction of differentiation of pluripotent stem cells (iPSCs) into male germ cells.^[^ ^194^ ^]^ They discovered that, in comparison to conventional 2D culture, the 3D‐DAM scaffold culture displayed significant expression of germ cell markers (VASA, DAZL, PLZF, STELLA, NANOS3), resulting in a more efficient production of haploid male germ cells. Furthermore, cell‐derived dECM scaffolds have been demonstrated to support the growth and differentiation of MSCs. Research indicates that stem cell‐derived dECM can aid in maintaining pluripotency and inducing differentiation into specific lineages, further emphasizing the versatility and potential of dECM hydrogels in tissue engineering and regenerative medicine.

**FIGURE 7:**
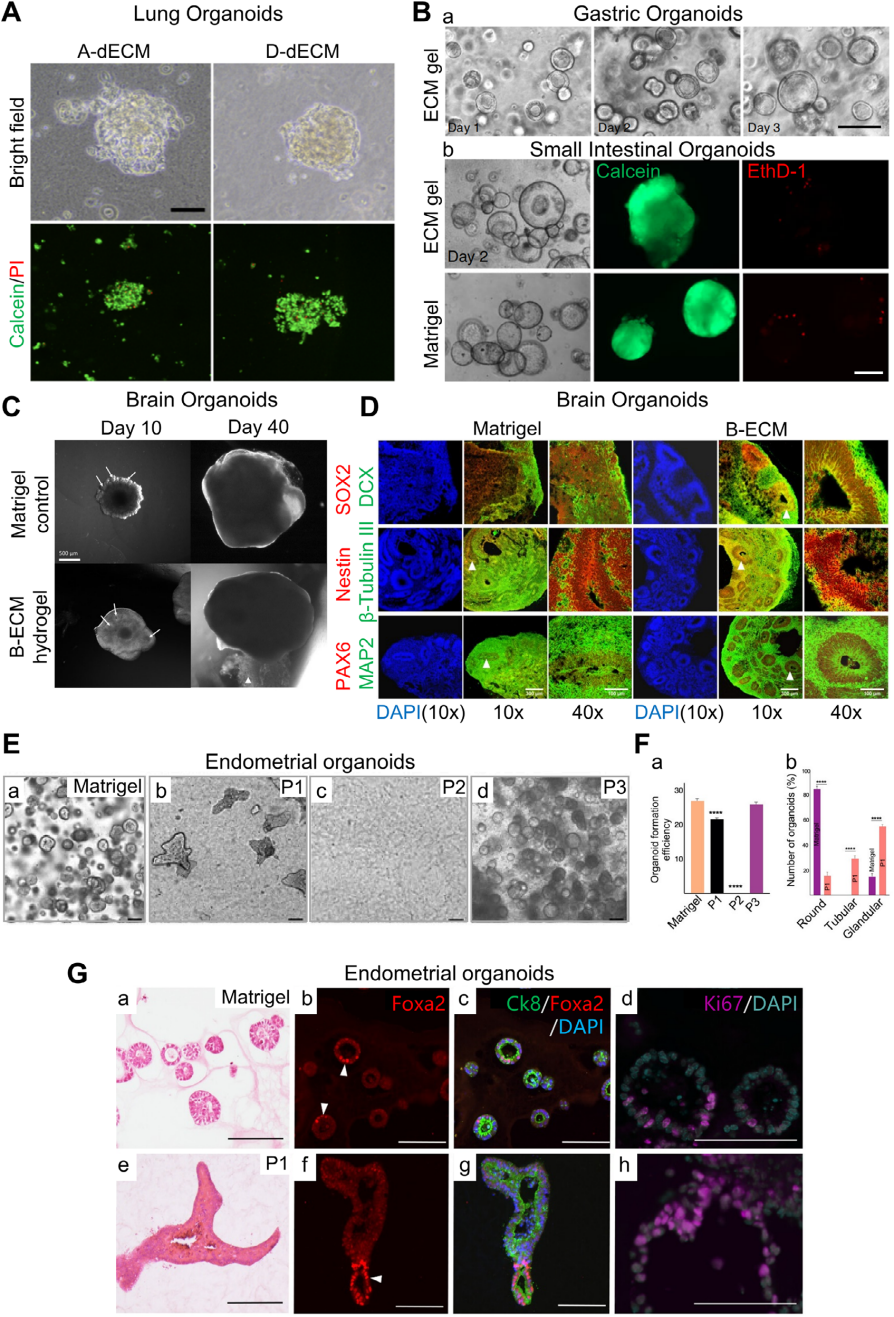
Applications of decellularized scaffolds in organoid culture. (A) Representative images of lung organoids encapsulated in A‐dECM and D‐dECM hydrogels and stained for calcein‐AM (green) and PI (red) on day 10. Reproduced under the terms of the CC‐BY 4.0 License.^[^ ^197^ ^]^ Copyright 2023, The Authors. (B) Human pediatric gastric enteroids in ECM gel (a), and forming mouse intestinal organoids per field of view at day 4 of culture in ECM gel and Matrigel over two passages (b). Reproduced under a Creative Commons Attribution 4.0 International License.^[^ ^195^ ^]^ Copyright 2019, The Authors. (C) Brain organoids on day 10 in Matrigel (top pictures) or B‐ECM hydrogel (bottom pictures). On day 40, no significant difference is apparent. White arrows at day 10 point to ventricular‐like zones located in the formed neuronal buds. White arrowhead at day 40 indicates residual B‐ECM hydrogel. Reproduced under the terms of the Creative Commons Attribution License.^[^ ^198^ ^]^ Copyright 2021, The Authors. (D) Tissue sections of brain organoids cultured in Matrigel (left panels) or B‐ECM hydrogel (right panels) for 40 days were immunostained for neuronal progenitor markers (SOX2, Nestin, PAX6) and mature neuronal markers (DCX, β‐Tubulin III, MAP2). DAPI was used as nuclear counterstaining. White arrowheads indicate ventricular like zones that were chosen for magnification. Reproduced under the terms of the Creative Commons Attribution License.^[^ ^198^ ^]^ Copyright 2021,The Authors. (E) Bright‐field images of mouse endometrial organoids in Matrigel and endometrial hydrogels (P1–P3, a–d). Reproduced under Creative Commons Attribution‐Non Commercial‐No Derivatives License 4.0 (CC BY‐NC‐ND).^[^ ^107^ ^]^ Copyright 2022, the Authors. (F) The organoid forming efficiency of mouse endometrial organoids in Matrigel and P1–P3 hydrogel (a); Percentages of organoid nos. representing round, tubular, and glandular‐shaped organoids in Matrigel versus P1 (b). Reproduced under Creative Commons Attribution‐Non Commercial‐No Derivatives License 4.0 (CC BY‐NC‐ND).^[^ ^107^ ^]^ Copyright 2022, the Authors. (G) H&E and immunostaining for Foxa2, Ck8, and Ki67 of organoids cultured in Matrigel and P1 hydrogel. Reproduced under Creative Commons Attribution‐Non Commercial‐No Derivatives License 4.0 (CC BY‐NC‐ND).^[^ ^107^ ^]^ Copyright 2022, the Authors.

Organoids are advanced in vitro model systems that mimic the complexity of organs with multicellular structures and functions. Traditionally, organoid cultures have depended on complex animal‐derived ECMs, like Matrigel and BME gel, which often possess undefined chemical compositions and exhibit poor tunability and reproducibility. Studies have demonstrated that liver ECM (LECM)‐derived hydrogels promote the proliferation of human bile duct organoids while preserving cholangiocyte‐like phenotypes. LECM hydrogels hold the potential to successfully replace tumor‐derived BME, thereby enhancing the capabilities of human cholangiocyte organoids. For example, Giobbe et al. showed that gels from decellularized porcine small intestine mucosa/submucosa could support the formation and growth of endoderm‐derived human organoids, including gastric, liver, pancreatic, and SI organoids. ECM hydrogels act as tools for direct human organoid derivation, cell growth with stable transcriptomic features, and for facilitating in vivo organoid delivery.^[^ ^195^ ^]^ Choi et al. cultured patient‐derived lung cancer organoids (LCOs) in conjunction with decellularized ECM (LudECM) hydrogels derived from lung tissue and Matrigel. They observed that LCOs grown in LudECM demonstrated significantly improved long‐term proliferation (>7 days). Importantly, the proliferation rates at 14 days were significantly different compared to those cultured in Matrigel, and LCOs grown on LudECM preserved the genetic alterations of the original cancer tissues.^[^ ^196^ ^]^ Kuzieoglu et al. engineered a hydrogel from decellularized bovine lung tissue ECM, which effectively preserved the morphology of patient‐derived lung organoids, offering a reproducible alternative for human lung tissue suitable for disease modeling.^[^ ^197^ ^]^ Simsa et al. discovered that hydrogels derived from decellularized adult porcine brain ECM (B‐ECM) served as scaffolds for brain organoids derived from human embryonic stem cells (hESCs). They could be further optimized for organoid growth by enhancing the preservation of proteins beyond collagen after decellularization.^[^ ^198^ ^]^ He et al. developed a novel method for primary hepatocyte organoid generation by jointly cultivating MSCs with hepatocytes on liver‐derived ECM hydrogels, offering potential for developing liver disease models and drug screening.^[^ ^199^ ^]^ Vermeulen et al. developed hydrogels from decellularized porcine immature testicular tissue (ITT) that successfully generated testicular organoids (TOs). They demonstrated that growth factors in TOs derived from decellularized ITT were better preserved, indicating the potential for enhanced restoration of reproductive capability.^[^ ^200^ ^]^ Okada et al. prompted the differentiation of neonatal mouse testicular cells into TOs using ECM hydrogels derived from ram testes, verifying that spermatogonial cells matured into post‐meiotic cells.^[^ ^201^ ^]^ Jamaluddin et al. fabricated three hydrogels from endometrial ECM and evaluated their quality based on organoid formation efficiency, shape, and size.^[^ ^107^ ^]^ After selecting P1 hydrogels, immunostaining and quantitative analysis of endometrial gland markers Foxa2 and proliferation marker Ki67 in organoids suggested that P1 hydrogels were capable of effectively replacing Matrigel for endometrial organoid culture. Kim et al. determined that gastrointestinal ECM hydrogels facilitated prolonged passaging and transplantation of organoids, producing organoids suitable for gastrointestinal disease modeling, drug development, and tissue regeneration by simulating the microenvironment of gastrointestinal tissues.^[^ ^202^ ^]^ These advancements demonstrate that extracellular matrices (ECMs) within decellularized scaffolds facilitate various functional applications of organoids, thereby contributing to the progress of tissue engineering technologies. Figure 7 summarizes the current application strategies based on decellularized scaffolds in organoid culture.

#### 6.2.2 Decellularized scaffolds in disease model construction and drug screening

The growing interest in disease pathology and the expanding biopharmaceutical market have highlighted the limitations of traditional 2D in vitro cell culture models, which fail to replicate the complex tissue architecture and cell interactions present in vivo. This has led to a push for alternative models that can better mimic human disease pathology and evaluate drug toxicity, with decellularized scaffolds offering a promising solution. Decellularized scaffolds retain the 3D structure and integrity of the original tissue, including its vascular networks, and can preserve pathological states such as fibrosis. This makes them ideal for creating disease models that more accurately reflect the human condition. For example, Zhang et al. demonstrated that decellularized fibrotic kidney scaffolds could be used to study the mechanisms of fibrogenesis and serve as both models for research and as potential donors for tissue engineering applications, which could be particularly beneficial for personalized drug development and pharmacotoxicology studies.^[^ ^203^ ^]^

Cancer research has also benefited from the use of decellularized scaffolds. Implanting cancer cells onto these scaffolds can create tumor models that closely replicate the interactions between cancer cells and the ECM. Ferreira's review in 2020 highlighted the progress and challenges of using dECM‐based biomaterials to mimic the tumor‐supportive matrix.^[^ ^204^ ^]^ Alabi et al. developed a 3D in vitro system using decellularized colons from both healthy and cancer‐prone mice to support human cancer cell growth, providing insights into how proteins within the tumor microenvironment might influence colorectal cancer progression.^[^ ^205^ ^]^

Additionally, matrix stiffness, an important regulator of tumor cell behavior, is being studied using dECM scaffolds. Lv et al. created 3D dECM scaffolds with varying stiffness to replicate the microenvironment of human breast tumors. By cultivating MDA‐MB‐231 breast cancer cells on these scaffolds, they investigated how matrix stiffness affects cell survival, apoptosis, and drug resistance.^[^ ^206^ ^]^ Such models are instrumental for preclinical drug screening and understanding tumor progression.

Decellularized scaffolds are not yet fully integrated into in vitro modeling, but their potential is clear. They represent a significant step forward in creating models that better represent tissue microenvironments, aiding in the understanding of disease mechanisms and the development of targeted therapies. As research progresses, these scaffolds are likely to become increasingly valuable tools for modeling health and disease, enhancing our understanding of pathological conditions, and driving the development of novel therapeutic strategies.

## 7 CONCLUDING REMARKS AND FUTURE PERSPECTIVES

Over the past two decades, dECM biomaterials have attracted substantial interest and research focus in the fields of tissue engineering and regenerative medicine. The therapeutic potential of these biomaterials, sourced from cells, tissues, and organs, is now being realized. dECM constitutes a complex and unique scaffold comprising structural proteins and GAGs, crucial for transmitting intrinsic physical and chemical signals, thus influencing cell behavior, tissue regeneration, vascular reconstruction, and homeostasis regulation. In the practical application of tissue engineering, compared with other engineered biological scaffolds, dECM scaffolds can effectively remove immunogenic cellular components and thus reduce potential adverse immune reactions after implantation. At the same time, the preservation of the 3D structure offers a stable physical framework and a biomimetic scaffold for signal binding and transmission for subsequent cell‐cell interactions and cell‐ECM interactions. Compared with synthetic polymers, dECM scaffolds facilitate diverse cellular functions due to the retention of functional biochemical constituents like cell adhesion molecules.

The significant advancements in decellularized scaffold biomaterials have underscored their numerous benefits and potential in preclinical and clinical scenarios, with dECM‐based products emerging as promising matrices for clinical adoption. Examples include AlloDerm (BioHorizons) and Oasis (Smith and Nephew) for skin regeneration, GraftJacket (Wright Medical) and Allopatch HD (MTF Sports Medicine) for musculoskeletal repair, and the decellularized pericardium‐based heart valve product CardioCel (Admedus IHS Inc.). Consequently, the application of these products has spurred further advancements in decellularization technologies.

Despite significant progress, several issues and challenges remain in the research and utilization of decellularized scaffolds. These challenges include the imperative to refine recellularization methods for optimized product design, a deeper comprehension of the mechanisms by which dECM modulates cellular behavior, and the achievement of mechanical properties on par with native tissues. The establishment of uniform sterilization and preservation protocols, in conjunction with advanced characterization techniques, is essential for the enhancement of clinical translation of decellularized tissue biomaterials and grafts. Furthermore, the development of chemotactic dECM biomaterials holds promise for actively facilitating tissue repair and regeneration, independent of exogenous cells and growth factors. Addressing these issues and challenges will expedite the actualization of decellularized scaffolds’ applications in tissue engineering and biomedicine, augmenting their utility in clinical and preclinical contexts, thereby expanding the scope of applications of decellularized scaffold materials across diverse disciplines.

## CONFLICT OF INTEREST STATEMENT

The authors declare no conflicts of interest.
